# Schwannoma development is mediated by Hippo pathway dysregulation and modified by RAS/MAPK signaling

**DOI:** 10.1172/jci.insight.141514

**Published:** 2020-10-15

**Authors:** Zhiguo Chen, Stephen Li, Juan Mo, Eric Hawley, Yong Wang, Yongzheng He, Jean-Philippe Brosseau, Tracey Shipman, D. Wade Clapp, Thomas J. Carroll, Lu Q. Le

**Affiliations:** 1 Department of Dermatology and; 2 Medical Scientist Training Program, University of Texas (UT) Southwestern Medical Center, Dallas, Texas, USA.; 3 Department of Pediatrics, Indiana University School of Medicine, Indianapolis, Indiana, USA.; 4 Department of Molecular Biology,; 5 Simmons Comprehensive Cancer Center, and; 6 Comprehensive Neurofibromatosis Clinic, UT Southwestern Medical Center, Dallas, Texas, USA.

**Keywords:** Genetics, Oncology, Signal transduction, Tumor suppressors

## Abstract

Schwannomas are tumors of the Schwann cells that cause chronic pain, numbness, and potentially life-threatening impairment of vital organs. Despite the identification of causative genes, including NF2 (Merlin), INI1/SMARCB1, and LZTR1, the exact molecular mechanism of schwannoma development is still poorly understood. Several studies have identified Merlin as a key regulator of the Hippo, MAPK, and PI3K signaling pathways; however, definitive evidence demonstrating the importance of these pathways in schwannoma pathogenesis is absent. Here, we provide direct genetic evidence that dysregulation of the Hippo pathway in the Schwann cell lineage causes development of multiple schwannomas in mice. We found that canonical Hippo signaling through the effectors YAP/TAZ is required for schwannomagenesis and that MAPK signaling modifies schwannoma formation. Furthermore, cotargeting YAP/TAZ transcriptional activity and MAPK signaling demonstrated a synergistic therapeutic effect on schwannomas. Our new model provides a tractable platform to dissect the molecular mechanisms underpinning schwannoma formation and the role of combinatorial targeted therapy in schwannoma treatment.

## Abstract 2

Canonical Hippo signaling through the effectors YAP/TAZ is required for the development of peripheral nervous system tumors of Schwann cells, and MAPK signaling modifies schwannoma formation.

## Graphical abstract

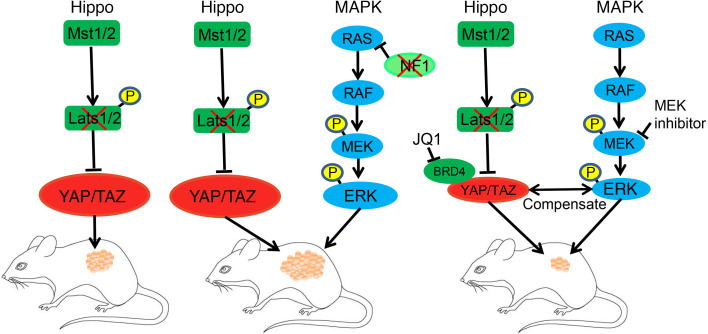

## Introduction

Schwannomas are tumors of Schwann cell origin found in patients with Neurofibromatosis Type 2 (NF2) and schwannomatosis, a recently characterized third major form of neurofibromatosis. In NF2, schwannomas can develop along peripheral, spinal, and cranial nerves, and they often develop along the eighth cranial nerve, resulting in bilateral acoustic (vestibular) schwannomas that cause hearing and balance problems. Patients with schwannomatosis lack this hallmark vestibular schwannoma but develop multiple schwannomas elsewhere throughout the body. In both NF2 and schwannomatosis, schwannomas can compress nearby nervous tissue, resulting in significant neurological impairment that results in pain, numbness, and weakness of the extremities; in schwannomatosis, the pain can be particularly debilitating and is often the first presenting symptom (1). Schwannomas most often occur sporadically outside any tumor-predisposition syndromes; however, they can undergo rare malignant transformation and cause life-threatening invasion of nearby vital organs (2–4).

Several genes including *NF2* (Merlin), *INI1/SMARCB1*, and the newly identified schwannomatosis-predisposing gene *LZTR1* are implicated in schwannoma development (5–8); however, their molecular roles are poorly defined. Using P0-Cre–induced *Nf2* gene deletion, Giovannini et al. first demonstrated that loss of *NF2* in the Schwann cell lineage was sufficient for schwannoma development and recapitulation of the human phenotype (9). It was subsequently shown that NF2 acts as a regulator of the Hippo pathway, a highly conserved kinase cascade initially discovered in *Drosophila* that regulates cell proliferation and organ size (10). Merlin activates the Hippo pathway by forming a complex with Hpo and Sav (orthologs of mammalian Mst1/2 and Sav1, respectively) in *Drosophila* (11, 12). The Mst1/2-Sav1 complex then phosphorylates and activates LATS1/2. In mammals, Sav1 recruits MST1/2 kinases to the plasma membrane for activation by upstream regulators. In parallel, Merlin recruits LATS1/2 kinases to the plasma membrane for phosphorylation and activation by MST1/2 kinases (13). Merlin can also modulate LATS1/2 activity through CRL4^DCAF1^ (14). Activated LATS1/2, in turn, phosphorylates and induces cytoplasmic retention and degradation of the transcription factors YAP and TAZ (15). In the absence of Hippo pathway signaling, YAP and TAZ translocate to the nucleus to form a transcriptional complex with TEAD1–4 and other transcription factors, including the bromodomain-containing protein 4 (BRD4), a member of the bromodomain and extraterminal (BET) family. This complex then initiates expression of target genes that stimulate proliferation and inhibit apoptosis (16, 17) (Supplemental Figure 1; supplemental material available online with this article; https://doi.org/10.1172/jci.insight.141514DS1).

While it has been hypothesized that NF2 mediates schwannoma development through Hippo pathway signaling, no direct evidence has been shown. Additionally, new evidence suggests that additional pathways may also be important for schwannoma development. In addition to its role in Hippo signaling, NF2 also regulates PI3K/mTOR/Akt, MAPK, RAS/RAF/ERK, RAC/CDC42/p21-activated kinases, and RhoGTPase family signaling pathways (18–24) (Supplemental Figure 1). Moreover, only about 60% of patients with schwannomas carry biallelic loss of *NF2* (25). Furthermore, although *INI1/SMARCB1* and *LZTR1* mutations show strong correlation with schwannomatosis, there is no direct evidence for their role in Hippo pathway signaling.

We reasoned that, if Hippo pathway dysregulation was required for schwannomagenesis, then mutation of the downstream kinases (i.e., LATS1/2) should also lead to schwannomagenesis. Previous studies have shown that KO of *Lats1/2* gene with a broad Schwann cell Cre, such as *Dhh-Cre*, results in direct malignant transformation and bypasses the benign schwannoma stage (26). Therefore, we hypothesized that a more restricted Schwann cell Cre might slow malignant transformation, enabling detection of the benign schwannoma stage. In this study, we generated a mouse model of schwannomatosis using *Hoxb7-Cre*–driven *Lats1/2* gene deletion. We provide direct genetic evidence that dysregulation of the Hippo pathway is necessary for schwannomagenesis and that MAPK signaling acts as a modifier for schwannoma formation. Moreover, pharmacological coinhibition of YAP/TAZ transcriptional activity and MAPK signaling shows a synergistic size reduction of mouse schwannoma. Our new model provides a framework to further clarify the molecular mechanisms of schwannoma development and identify potential therapeutic targets.

## Results

### Hippo pathway inactivation in Hoxb7^+^ lineage cells results in formation of multiple schwannomas.

**Figure 1:**
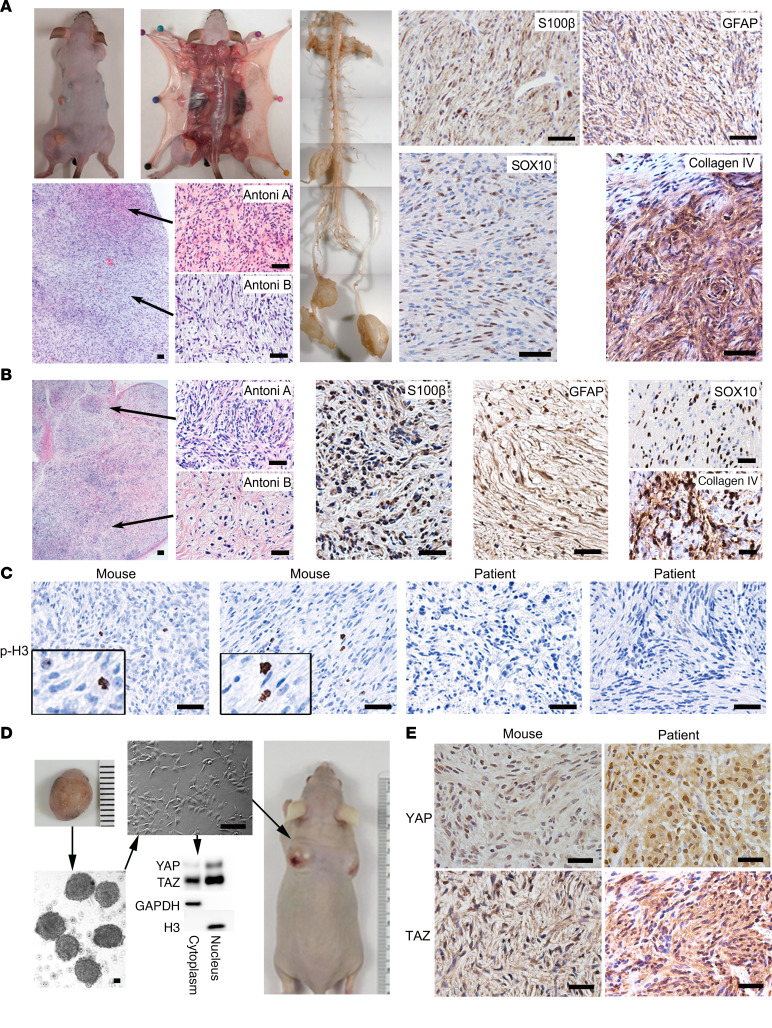
Hippo pathway inactivation in Hoxb7^+^ lineage cells results in multiple schwannoma formation. (**A**) Dissection and histological characterization of mouse schwannoma: H&E and IHC of Schwann cell markers (S100β and GFAP), a neural crest marker (Sox10), and collagen IV. (**B**) H&E and IHC of S100β, GFAP, SOX10, and Collagen IV on human schwannoma tissue sections. (**C**) IHC of phospho-Histone H3 on mice and human schwannoma tissue sections. (**D**) Mouse schwannomas were harvested, and tumorsphere cell culture was performed. Tumorspheres were then seeded to fibronectin-coated plates for monolayer culture. Both cytoplasmic and nuclear fractions isolated from these monolayer cultured cells were analyzed by Western blotting. The tumor cells were injected into nude mice s.c. (*n* = 6). (**E**) IHC of YAP and TAZ on mouse and human schwannoma tissue sections. Scale bars: 50 μm.

We previously showed that *Hoxb7-Cre* was a more restricted Schwann cell Cre and that the Hoxb7^+^ lineage comprises a subset of Schwann cells in peripheral nerves with tumorigenic potential (27). In order to determine whether Hippo pathway inactivation is sufficient for schwannomagenesis, we crossed the *Hoxb7-Cre* mice with *Lats1^fl/fl^;Lats2^fl/fl^* mice to obtain the *Hoxb7-Cre;Lats1^fl/fl^;Lats2^+/+^, Hoxb7-Cre;Lats1^+/+^;Lats2^fl/fl^, Hoxb7-Cre;Lats1^fl/+^;Lats2^fl/+^* (hereafter called *H7;Lats1/2mut2*), *Hoxb7-Cre;Lats1^fl/fl^;Lats2^fl/+^, Hoxb7-Cre;Lats1^fl/+^;Lats2^fl/fl^* (hereafter called *H7;Lats1/2mut3*), and *Hoxb7-Cre;Lats1^fl/fl^;Lats2^fl/fl^* (hereafter called *H7;Lats1/2mut4*). *H7;Lats1/2mut2* mice did not develop tumors, and *H7;Lats1/2mut4* mice were embryonic lethal. Only *H7;Lats1/2mut3* mice gave rise to multiple masses in skin, soft tissue, and dorsal root ganglions (DRG) (Supplemental Table 1 and 2) (Figure 1A). Further characterization of these well-circumscribed masses indicated a mixture of hypercellular (Antoni A) areas and hypocellular (Antoni B) areas, diffuse/strong expression of Schwann cell markers S100β and GFAP, neural crest lineage marker SOX10, and abundant pericellular collagen type IV (Figure 1A). These results recapitulate the histology of human schwannoma (Figure 1B) and meet the pathologic diagnostic criteria for schwannoma (28). Some of these tumors underwent malignant transformation, as indicated by phosphohistone H3 (p-H3, a mitosis marker) staining, consistent with increased mitotic activity and allograft assays in nude mice (Figure 1, C and D).

If the Hippo pathway is crucial for schwannoma development, inactivation of *Lats1/2* should result in YAP/TAZ dephosphorylation and nuclear localization. As expected, YAP/TAZ was mainly localized to nuclei of cultured mouse tumor cells (*Hoxb7-Cre;Lats1^fl/+^;Lats2^fl/fl^*) (Figure 1D). In addition, we also observed nuclear staining of YAP/TAZ in both human and mouse schwannoma tissues (Figure 1E). Altogether, these data suggest that dysregulation of Hippo pathway signaling is sufficient for schwannomagenesis.

### Lats1 or Lats2 loss of heterozygosity in H7;Lats1/2mut3 mice is required for schwannoma development.

**Figure 2:**
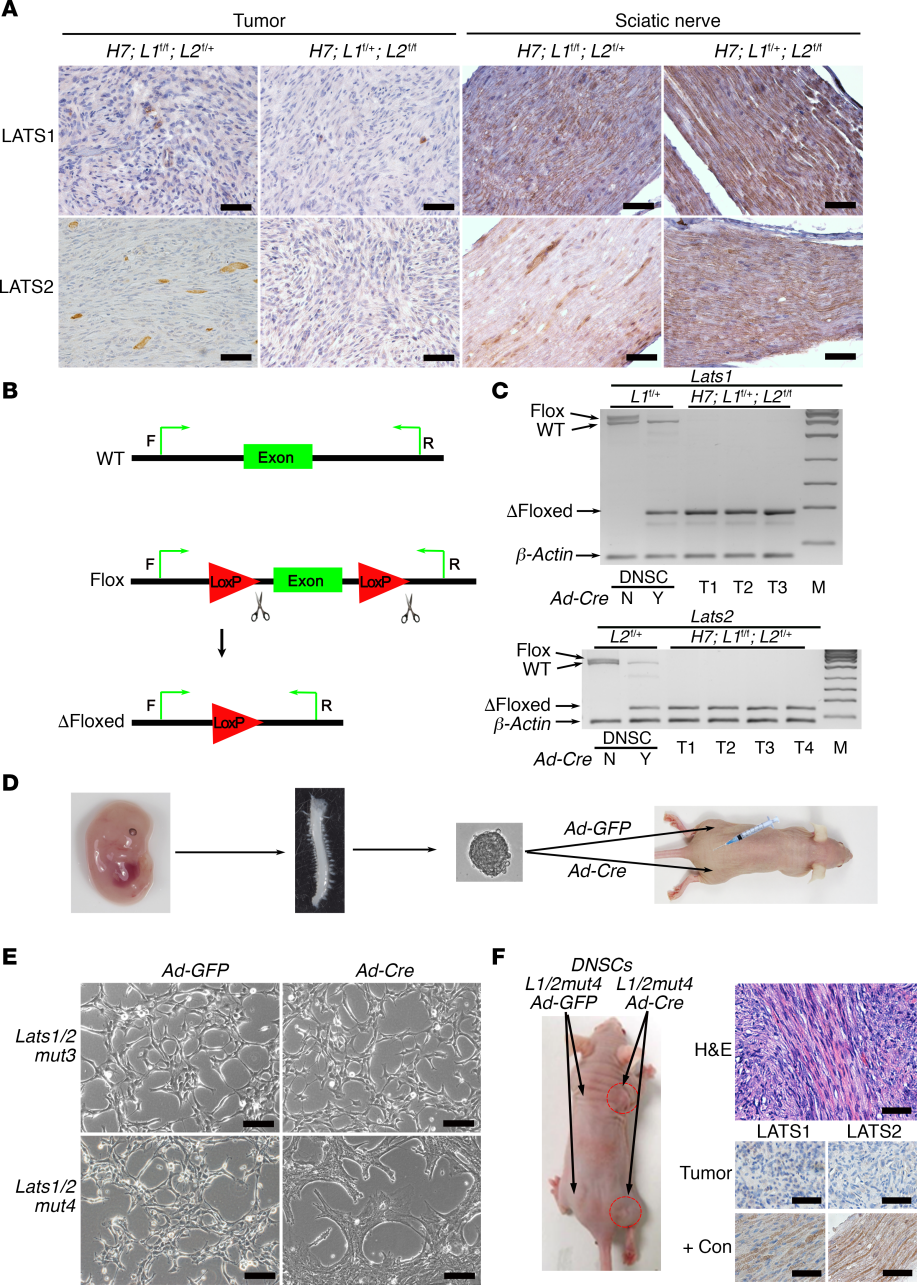
*Lats1* or *Lats2* loss of heterozygosity in *H7;Lats1/2mut3* mice is required for schwannoma development. (**A**) IHC of LATS1 and LATS2 on schwannoma and sciatic nerve sections from *H7;Lats1/2mut3* mice. (**B**) Diagram of PCR primer design for detecting the WT, Flox, or Δfloxed alleles of *Lats1* or *Lats2*. (**C**) Gel electrophoresis of DNSC and tumor PCR products. β-Actin was used as an internal control (*n* = 3–4). Y, Ad-Cre infected; N, no Ad-Cre infected; T, tumors; M, DNA marker. (**D**) Diagram of experimental design for testing tumor formation potential of E13.5 DNSCs in vivo. (**E**) Morphology of E13.5 DNSCs (*Lats1/2mut*) infected with *Ad-GFP* or *Ad-Cre*. (**F**) Left: Gross picture of nude mice injected with E13.5 DNSCs (*Lats1/2mut4*-*Ad-GFP*) (left side) or E13.5 DNSCs (*Lats1/2mut4*-*Ad-Cre*) (right side, red circles) (*n* = 4). Right: H&E histology (top) and IHC (bottom) for LATS1 and LATS2 in *Lats1/2mut4*-*Ad-Cre* tumor. *L1/2mut4*, *Lats1/2mut4*; +Con, positive control; *H7*, *Hoxb7-Cre*; *L1*, *Lats1*; *L2*, *Lats2*. Scale bars: 50 μm.

*H7;Lats1/2mut3* mice each developed an average of 15.4 tumors. While Hoxb7 lineage cells comprise only a small subset of Schwann cells in peripheral nerves, the total Hoxb7^+^ cell number is still considerable (27). Therefore, if triallelic loss of *Lats1/2* (*Lats1^fl/fl^;Lats2^fl/+^* or *Lats1^fl/+^;Lats2^fl/fl^*) in the Hoxb7^+^ cell lineage is sufficient for schwannomagenesis, we would expect to see a greater number of tumors formed. We hypothesized that loss of heterozygosity of the last *Lats1* or *Lats2* WT allele in *H7;Lats1/2mut3* mice is required for schwannomagenesis. To test our hypothesis, we first analyzed LATS1/2 expression in these tumors. Immunohistochemical staining showed complete absence of LATS1/2 expression in *H7;Lats1/2mut3* tumors (Figure 2A), suggesting loss of the remaining allele. Next, we designed genotyping PCR primers to amplify WT, flox, and Δfloxed *Lats1* and *Lats2* alleles (Figure 2B). As a positive control, we used E13.5 *Lats1^fl/+^;Lats2^fl/+^* DRG/nerve root sphere cells (DNSCs). We previously showed that DNSCs contain embryonic Schwann cell precursors with tumorigenic potential that give rise to Schwann cell tumors when cultured and transplanted into mice (29). We infected these DNSCs with *Adenovirus-CMV-Cre-EGFP* (*Ad-Cre*) to delete the floxed *Lats1* and *Lats2* alleles. Using PCR amplification, we were able to detect WT, flox, and Δfloxed *Lats1* and *Lats2* alleles in infected or uninfected DNSCs (Figure 2C). Next, we genotyped primary tumor cell lines derived from *H7;Lats1/2mut3* mouse schwannomas (Figure 2C). We were unable to detect the WT allele of *Lats1* or *Lats2*, further demonstrating loss of heterozygosity.

We previously demonstrated the value of E13.5 DNSCs for preclinical drug screening and gene editing (27, 29). Therefore, we tested whether 3- or 4-allele ablation of *Lats1* and *Lats2* in E13.5 DNSCs was sufficient for tumorigenesis in nude mice. We infected E13.5 DNSCs (*Lats1/2mut3* and *Lats1/2mut4*) with *Ad-Cre* and injected them into the sciatic nerve (SN) and s.c. tissue of nude mice. *Adenovirus-CMV-EGFP* (*Ad-GFP*) transduction was used as control (Figure 2D). *Ad-Cre* transduced *Lats1/2mut3* DNSCs did not undergo obvious morphological change. However, *Ad-Cre* transduced *Lats1/2mut4* DNSCs (Supplemental Figure 2) underwent a striking morphological change from small and thin to wide-spreading and flat after *Ad-Cre* transduction (Figure 2E). When injected into SN and s.c. tissue of nude mice, only *Lats1/2mut4-Ad-Cre* DNSCs gave rise to tumors (Figure 2F). Our results show that loss of heterozygosity of the residual WT *Lats1* or *Lats2* allele in *H7;Lats1/2mut3* mice is required for schwannoma development. However, *NF2* loss in the Schwann cell lineage could also lead to schwannoma development, and although the likelihood is very low, it is still possible that schwannomagenesis may result from *NF2* loss/downregulation as a result of *Lats1/2* loss. To exclude this possibility, we performed immunostaining of NF2 in mouse schwannoma. We observed that NF2 was highly expressed in mouse schwannoma (Supplemental Figure 3A). In addition, Western blot analysis also showed that NF2 and other Hippo pathway components upstream of LATS1/2, including MST1/2, SAV1, and MOB1, were also expressed (Supplemental Figure 3B), which suggests that *NF2* was not lost and was not involved in schwannomagenesis of *H7;Lats1/2mut3* mice. These data suggest that both *Lats1* and *Lats2* loss are required for schwannomagenesis.

### Loss of YAP or TAZ alone does not inhibit schwannoma proliferation.

**Figure 3:**
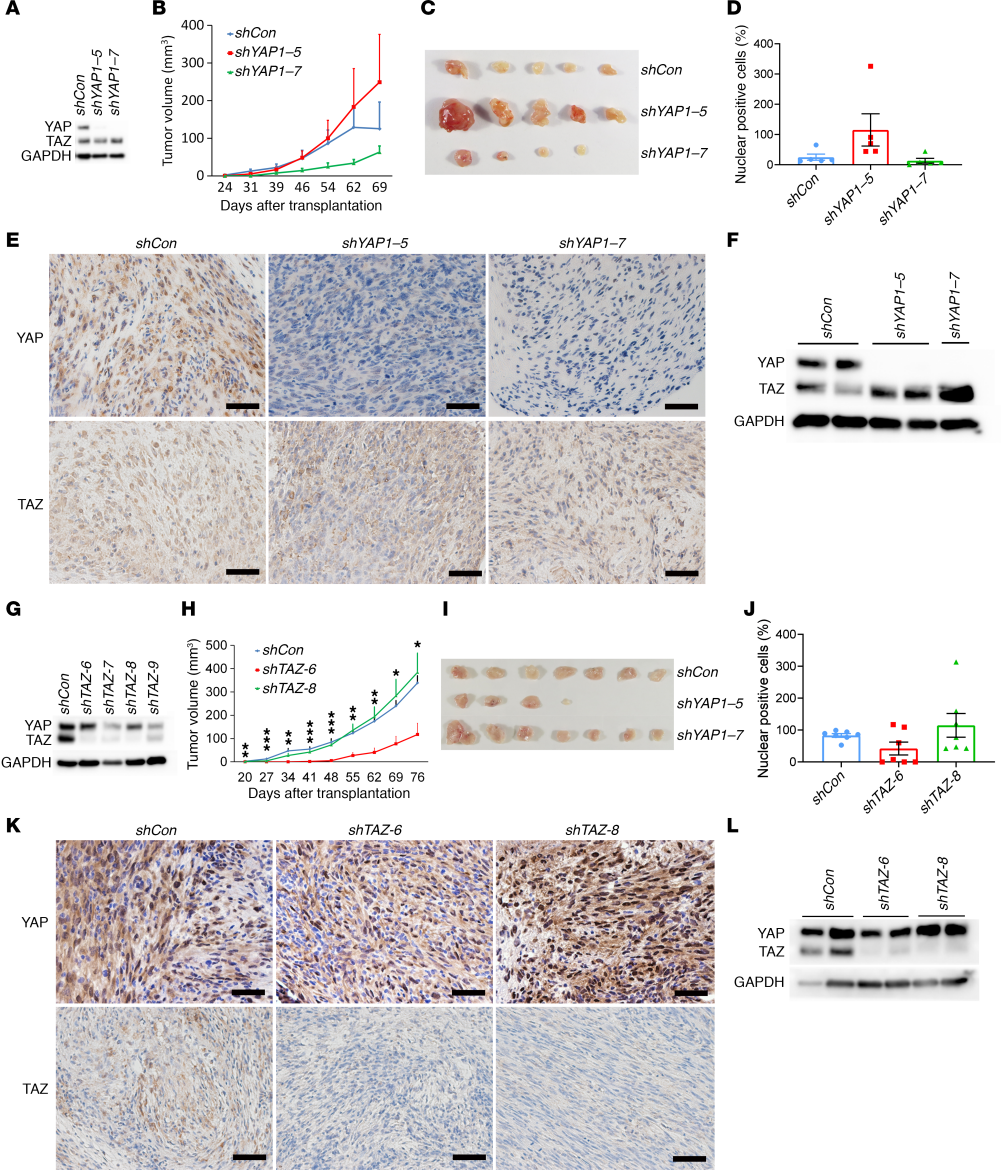
Loss of *YAP* or *TAZ* alone does not inhibit schwannoma proliferation. (**A**) Western blot analysis of YAP and TAZ protein level in shControl, shYAP-5, and shYAP-7 transduced tumor cells (*Hoxb7-Cre;Lats1^fl/+^;Lats2^fl/fl^)*. shCon, shControl. (**B**) Tumor volume of shControl, shYAP-5, and shYAP-7 transduced tumor cells after transplantation into nude mice (*n* = 5/group). (**C**) Gross picture of tumors from the experimental endpoint in **B** (*n* = 5/group). (**D**) Average weight of excised tumors from **C** (*n* = 5/group). (**E**) Representative images of allografted tumor sections stained for YAP and TAZ. (**F**) Western blot analysis of YAP and TAZ protein level in cultured tumor–derived cells. (**G**) Western blot analysis of YAP and TAZ protein level in shControl, shTAZ-6, shTAZ-7, shTAZ-8, and shTAZ-9 tumor cells. (**H**) Tumor volume of shControl, shTAZ-6, and shTAZ-8 schwannomas in nude mice (*n* = 7/group). (**I**) Gross picture of tumors from the experimental endpoint in **H** (*n* = 7/group). (**J**) Average weight of excised tumors from **I** (*n* = 7/group). (**K**) Representative images of tumor sections stained for YAP and TAZ. (**L**) Western blot analysis of YAP and TAZ protein levels in cultured tumor cells derived from tumors in **I**. Scale bars: 50 μm. One-way ANOVA was applied to evaluate statistical significance in **B**, **D**, **H**, and **J**. All statistics are represented as the mean ± SEM. *P < 0.05, **P < 0.01, ***P < 0.001.

LATS1/2 are negative regulators of YAP/TAZ in canonical Hippo signaling (Supplemental Figure 1). Given that YAP exerts a stronger influence on multiple cellular processes than TAZ (30), we hypothesized that loss of YAP would impair schwannoma growth. We first tested whether knockdown of *YAP* transcript would inhibit the proliferation of tumor cells by employing noninducible small hairpin RNAs (shRNAs). Tumor cells derived from *H7;Lats1/2mut3* mice were transduced with lentivirus harboring either scrambled shRNA (pLKO.1-shCon) or YAP shRNAs (pLKO.1-shYAP-5 and pLKO.1-shYAP-7). Transduction of shYAP-5 and shYAP-7 resulted in complete knockdown of YAP protein (Figure 3A). To study the influence of YAP on the tumorigenic capacity of these cells, we performed s.c. injection of shCon, shYAP-5, and shYAP-7 tumor cells into nude mice. Unexpectedly, loss of YAP did not significantly impair schwannoma growth in vivo, with results being highly variable depending on the specific shRNA and experimental replicate (Figure 3, B–D). One explanation is that the puromycin-selected cell populations may have been contaminated with puromycin-resistant shYAP-negative cell clones. Another possibility is functional redundancy of TAZ. In order to rule out contamination, we analyzed the expression of YAP and TAZ in these tumors and tumor-derived cells. We found persistent loss of YAP expression in both shYAP1–5 and shYAP1–7 tumors and tumor-derived cells (Figure 3, E and F), while TAZ expression remained present. Similarly, we tested whether ablation of YAP alone could inhibit schwannoma proliferation in *PostnCre;Nf2^fl/fl^;YAP^fl/fl^* mice. Previous studies have shown that Postn-Cre drives reporter gene expression in Schwann cell progenitors starting at E10 (31, 32). We found that YAP deletion did not affect tumor burden or lifespan (Supplemental Figure 4, A–C). These results suggest that YAP alone is not essential for schwannoma development and that TAZ may compensate for YAP loss of function.

Next, we dissected the function of TAZ in schwannomagenesis using the same pLKO.1-shRNA system. Four different pLKO.1-shTAZ lentiviruses were constructed and transduced into tumor cells (*Hoxb7-Cre;Lats1^fl/+^;Lats2^fl/fl^*). We chose pLKO.1-shTAZ-6a and pLKO.1-shTAZ-8f for further experiments based on degree of protein knockdown (Figure 3G). After s.c. injection of tumor cells into nude mice, we observed similar results to the YAP knockdown. Knockdown of TAZ did not result in significantly reduced tumor growth, with highly varying results dependent on the shRNA used and experimental replicate (Figure 3, H–J). IHC and Western blot confirmed loss of TAZ protein in transplanted tumors and tumor-derived cells (Figure 3, K and L). Taken together, these results suggest that TAZ alone is also not essential for schwannoma development due to potential functional redundancy with YAP.

### YAP/TAZ gene dosage determines the tumor burden and survival.

As key downstream effectors of the Hippo pathway, YAP and TAZ may have both distinct and overlapping functions (30, 33). This potential redundancy may enable schwannomas to tolerate loss of either YAP or TAZ. In order to test whether simultaneous ablation of both *YAP* and *TAZ* in Hoxb7^+^ lineage cells prevents or rescues the tumor phenotype of *H7;Lats1/2mut3* mice, we crossed *H7;Lats1/2mut3* mice with *YAP^fl/fl^;TAZ^fl/fl^* mice to obtain *H7;Lats1/2mut3;YAP/TAZmut4* (*YAP^fl/fl^;TAZ^fl/fl^*), *H7;Lats1/2mut3;YAP/TAZmut3* (including *YAP^fl/fl^;TAZ^fl/+^* and *YAP^fl/+^;TAZ^fl/fl^* ), and *H7;Lats1/2mut3;YAP/TAZmut2* (including *YAP^fl/+^;TAZ^fl/+^*, *YAP^fl/fl^;TAZ^+/+^* and *YAP^+/+^;TAZ^fl/fl^*) mice.

**Figure 4:**
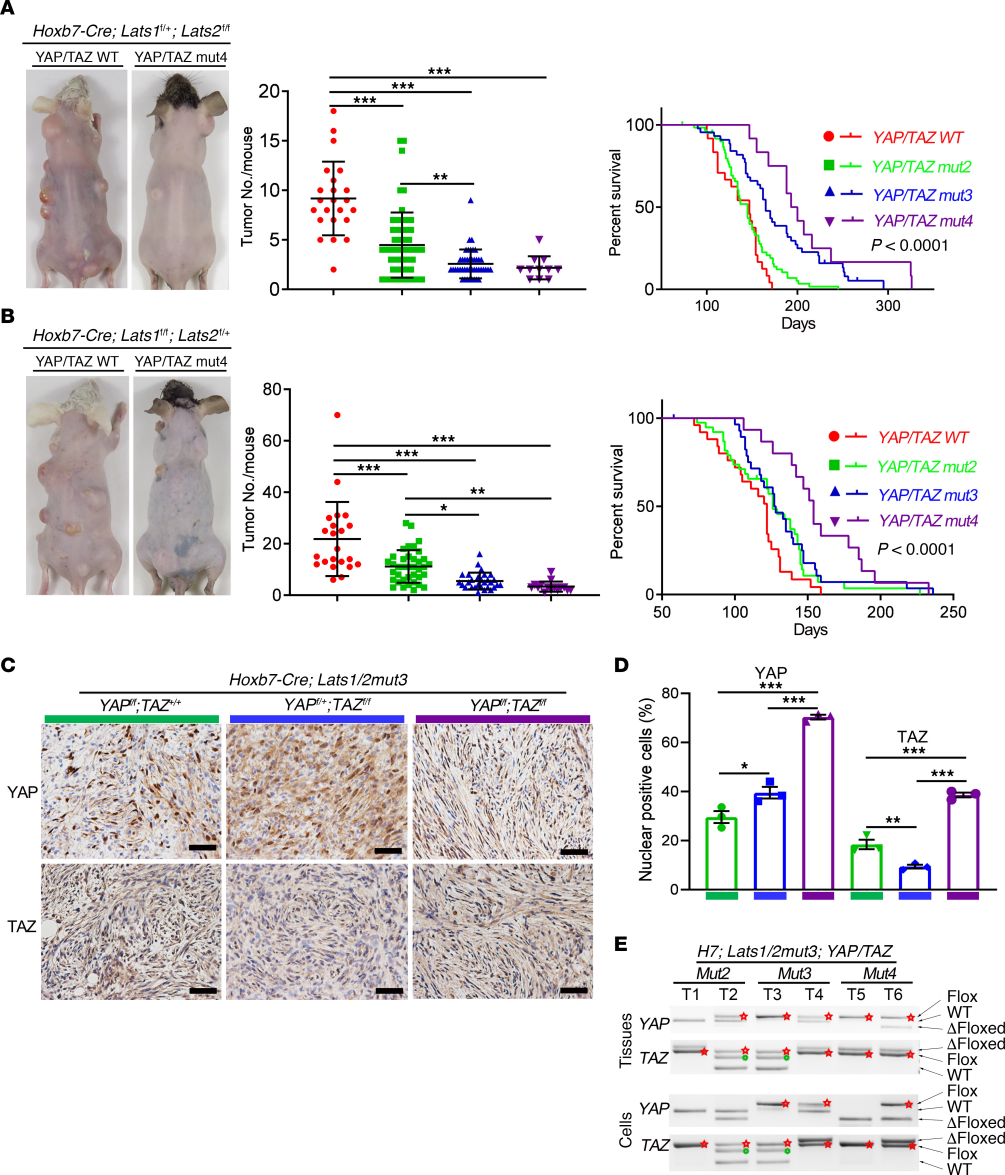
YAP/TAZ gene dosage determines the tumor burden and survival. (**A** and **B**) Representative pictures of tumor-burdened mice (left panel). Scatter plot of the total number of palpable tumors in each mouse (middle panel). Kaplan-Meier plot illustrating the survival curve among the groups with corresponding genotypes (right panel) for *Hoxb7-Cre;Lats1^fl/+^;Lats2^fl/fl^* (**A**) and *Hoxb7-Cre;Lats1^fl/fl^;Lats2^fl/+^* (**B**) mice. *H7;Lats1^fl/+^;Lats2^fl/fl^;YAP/TAZmut4*, *n* = 12. *H7;Lats1^fl/+^;Lats2^fl/fl^;YAP/TAZmut3*, *n* = 44. *H7;Lats1^fl/+^;Lats2^fl/fl^;YAP/TAZmut2*, *n* = 60. *H7;Lats1^fl/+^;Lats2^fl/fl^;YAP/TAZ^WT^*, *n* = 24. *H7;Lats1^fl/fl^;Lats2^fl/+^;YAP/TAZmut4*, *n* = 15. *H7;Lats1^fl/fl^;Lats2^fl/+^;YAP/TAZmut3*, *n* = 29. *H7;Lats1^fl/fl^;Lats2^fl/+^;YAP/TAZmut2*, *n* = 39. *H7;Lats1^fl/fl^;Lats2^fl/+^;YAP/TAZ^WT^*, *n* = 25. (**C**) IHC of YAP and TAZ on tumor sections. (**D**) Quantification of IHC in **C** (*n* = 3/group). (**E**) Genotyping of tumor tissues (upper panel) and tumor-derived cell lines (lower panel). Red star, intact floxed allele. Green dot, nonspecific amplification. Scale bars: 50 μm. One-way ANOVA with Tukey’s test for multiple comparisons were applied to evaluate statistical significance in **A**, **B**, and **D**. Log-rank statistical test was employed in **A** and **B**. Statistics are represented as the mean ± SEM. *P < 0.05, **P < 0.01, ***P < 0.001.

All of the *H7;Lats1/2mut3;YAP/TAZmut* mice were viable, fertile, and normal in size, with no gross behavioral or physical abnormalities except for multiple schwannomas with 100% penetrance. Compared with *H7;Lats1/2mut3* mice, *H7;Lats1/2mut3;YAP/TAZmut4*, *H7;Lats1/2mut3;YAP/TAZmut3,* and *H7;Lats1/2mut3;YAP/TAZmut2* mice exhibited a YAP/TAZ dosage–dependent increase in time to tumor onset and decrease in tumor number (Figure 4, A and B). We also observed significantly improved survival in *H7;Lats1/2mut3;YAP/TAZmut3* and *H7;Lats1/2mut3;YAP/TAZmut4* mice compared with *H7;lats1/2mut3* (Figure 4, A and B) (Supplemental Table 1 and 2). Interestingly, while *H7;Lats1/2mut4* mice died in utero, several *H7;Lats1/2mut4;YAP/TAZmut4* mice survived until P10–P20, which suggests YAP/TAZ deletion partially rescued not only the tumorigenic phenotype in *H7;Lats1/2mut3* mice, but also the survival of *H7;Lats1/2mut4* mice.

While *H7;Lats1/2mut3;YAP/TAZ mut4* mice showed significantly reduced tumor burden, they still developed schwannomas. To confirm whether *YAP/TAZ* were completely ablated in the tumors of these mice, we performed immunostaining of YAP and TAZ. Surprisingly, strong YAP/TAZ signal was still detectable in these tumors, suggesting incomplete ablation of the *YAP/TAZ* allele (Figure 4, C and D). Further genotyping confirmed that at least 1 intact *YAP* or *TAZ* allele was present in all tumor and tumor-derived cell lines, indicating that a subpopulation of tumor cells may have escaped Cre-mediated recombination, resulting in delayed tumor formation (Figure 4E). These data suggest that *YAP/TAZ* gene dosage determines tumor burden and survival, and that a single allele of *YAP* or *TAZ* is sufficient for schwannoma formation. Immunohistochemical analysis showed increased phospho-ERK in tumors from *Hoxb7;Lats1/2mut3;YAP/TAZmut4* compared with *Hoxb7;Lats1/2mut3* mice, indicating that YAP/TAZ ablation activated the MAPK pathway (Supplemental Figure 5).

### Canonical Hippo signaling through YAP/TAZ is required for schwannomagenesis.

**Figure 5:**
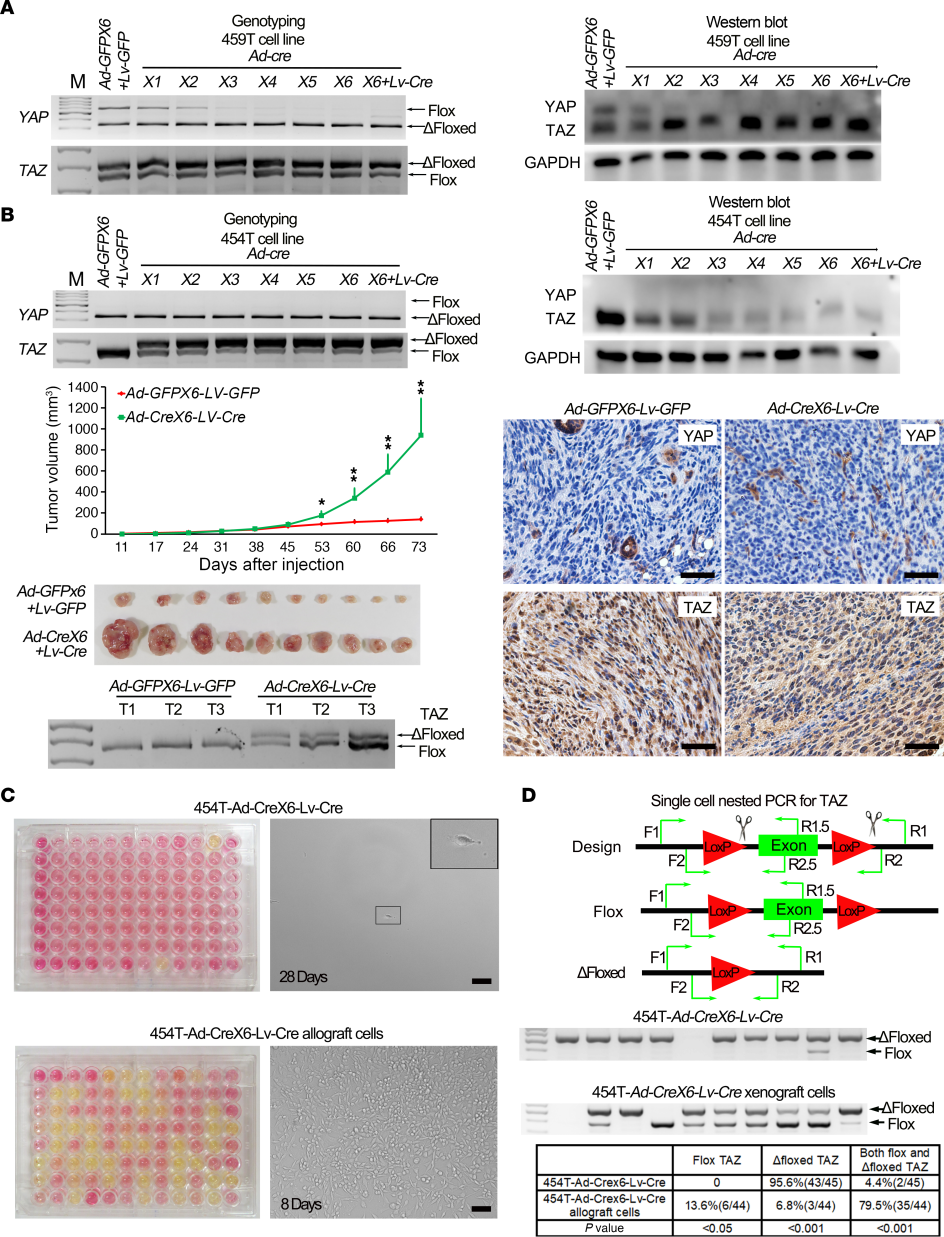
Canonical Hippo signaling through YAP/TAZ is required for schwannomagenesis. (**A**) Genotyping (left) and Western blot (right) of YAP and TAZ in 459T cells serially infected with *Ad-GFP/Lv-GFP* or *Ad-Cre/Lv-Cre*. (**B**) Genotyping (left top panel) and Western blot (right top panel) of YAP and TAZ in 454T cells serially infected with *Ad-GFP* or *Ad-Cre/Lv-Cre*; tumor volume of 454T-*Ad-GFPX6-Lv-GFP* and 454T-*Ad-CreX6-Lv-Cre* schwannoma tumor in nude mice (middle left panel) (*n* = 10/group); gross picture of tumors from experimental endpoint (lower left panel) (*n* = 10/group); IHC (lower right panel) and genotyping analysis of TAZ (lower left panel) in transplanted nude mice tumor tissue and its derived tumor cell lines. (**C**) Single cell clonal analysis of 454T-*Ad-Crex6-Lv-Cre* cells (upper panel) and their transplanted tumor–derived cells (lower panel). Yellow cell culture media indicates the proliferation of single cell clone; pictures were taken at 28 days (454T-*Ad-Crex6-Lv-Cre* cells) and 8 days (transplanted tumor–derived cells). (**D**) Single cell nested PCR for *TAZ*. Diagram of PCR primer design for detecting the flox or Δfloxed allele of *TAZ* in single cell level. F1, R1, and R1.5 primers were used for the first PCR. F2, R2, and R2.5 primers were used for the second PCR (upper panel). Representative pictures of gel electrophoresis for single cell nested PCR products (middle panel). Quantification of flox and Δfloxed alleles of *TAZ* from 454T-*Ad-Crex6-Lv-Cre* cells and their allograft-derived cells (lower panel). 454T-*Ad-Crex6-Lv-Cre* cells, *n* = 45. 454T-*Ad-Crex6-Lv-Cre* allograft cell, *n* = 44. First lane, DNA marker; empty lane, failure to detect the signal. Each lane represents a single cell. Scale bars: 50 μm. Two-tailed Student’s *t* test was applied to evaluate statistical significance in **B**. Statistics are represented as the mean ± SEM. *P < 0.05, **P < 0.01.

To investigate whether YAP/TAZ are critical for schwannomagenesis, we first tested whether or not *YAP/TAZ* could be completely ablated. We cultured tumor cell lines derived from *H7;Lats1/2mut3;YAP/TAZmut4* mice — 459T cells — and transduced them with *Ad-Cre* to ablate residual *YAP* and *TAZ*. We optimized the virus concentration to minimize cytotoxicity and ensure at least 95% gene delivery for each transduction by monitoring GFP expression. We found that a single round of *Ad-Cre* transduction resulted in only partial recombination of the *YAP* loxP sites (Figure 5A). We then tested up to 6 rounds of serial infections. Surprisingly, only *YAP* was ablated after multiple infections. The *TAZ* flox allele was present even after 6 rounds of serial infections with *Ad-Cre* (Figure 5A). Furthermore, addition of *Lentivirus-Cre* did not result in recombination of the *TAZ* allele. We observed similar results in multiple *H7;Lats1/2mut3;YAP/TAZmut4* tumor–derived cell lines.

To test whether complete *YAP/TAZ* ablation inhibits the proliferation of schwannoma in vivo, we chose a *H7;Lats1/2mut3;YAP/TAZmut4*-derived tumor cell line, 454T, harboring complete *YAP* loss and 2 intact *TAZ* alleles. After 6 rounds of *Ad-Cre* transduction and 1 round of *Lenti-Cre* infection (*Ad-Crex6-Lv-Cre*), only a small portion of *TAZ* alleles remained intact (Figure 5B). Next, we injected these cells into nude mice s.c. Cells infected with 6 rounds of *Adenovirus-GFP* (*Ad-GFP*) and 1 of *Lentivirus-GFP* (*Lenti-GFP*) were injected s.c. as a control (*Ad-GFPX6-Lv-GFP*). We found that partial ablation of *TAZ* resulted in enhanced tumor growth (Figure 5B). Immunostaining and genotyping confirmed that *TAZ* was still highly expressed in transplanted tumors, suggesting positive clonal selection based on *TAZ* status (Figure 5B and Supplemental Figure 6A). We also observed similar results using a different tumor cell line (Supplemental Figure 6, B and C). Given that the genotyping PCR results were based on a mixture of cells, we performed flow cytometry to isolate and seed single cells into each well of 96-well plates to obtain single cell clones from 454T-Ad-Crex6-Lv-Cre cells and their allografted tumors. After 18 days in culture, only 2 of the 96 sorted 454T-Ad-Crex6-Lv-Cre cells exhibited clonal expansion (Figure 5C). However, 42 of the 96 454T-Ad-Crex6-Lv-Cre allograft–derived cells exhibited clonal expansion (Figure 5C). We carefully searched individual wells of nonexpanding clones and observed that single cells isolated from 454T-Ad-Crex6-Lv-Cre cells remained nonproliferative, even at 28 days in culture (Figure 5C).

We then designed nested PCR primers to detect *TAZ* status on a single cell level. Consistent with our clonal analysis, we found that 95.6% (43 of 45) of single 454T-Ad-Crex6-Lv-Cre cells had complete recombination of the remaining *TAZ* allele, while the remaining 4.4% (2 of 45) carried both a flox and Δfloxed *TAZ*. For sorted 454T-Ad-Crex6-Lv-Cre allograft cells, only 6.8% (3 of 44) of single cell clones exhibited recombination of the remaining *TAZ* allele, while 79.5% (35 of 44) carried both a flox and Δfloxed *TAZ* allele and 13.6% (6 of 44) carried only floxed *TAZ* alleles (Figure 5D). Taken together, our data show that complete ablation of *YAP/TAZ* inhibits the proliferation of tumor cells, providing definitive evidence that canonical Hippo signaling is necessary and sufficient for schwannoma development. In addition, our studies demonstrate a need to develop inhibitors that simultaneously target both YAP and TAZ or their interaction for transcriptional activity.

### The MAPK pathway is a modifier of schwannomagenesis.

**Figure 6:**
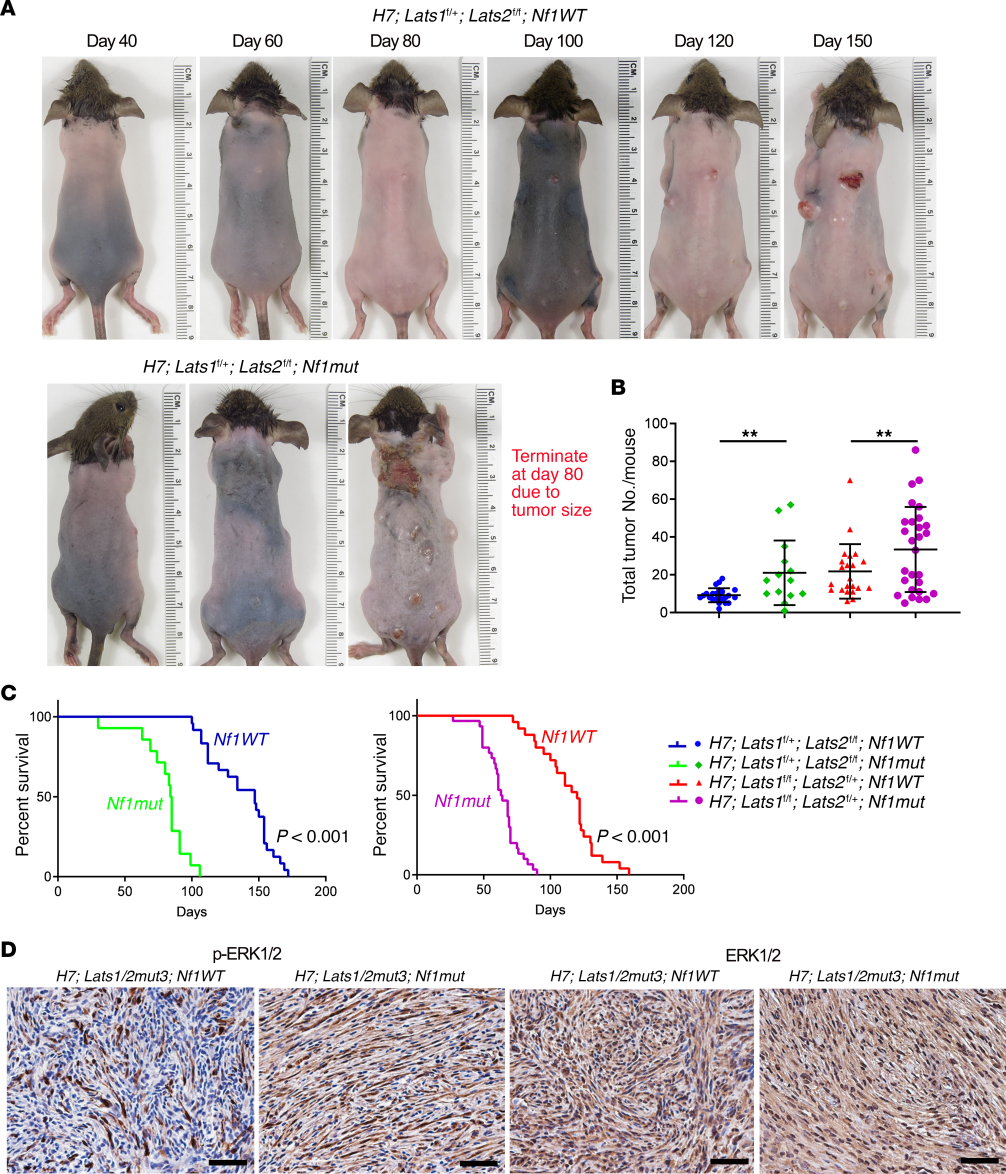
The MAPK pathway is a modifier of schwannomagenesis. (**A**) Representative pictures of *H7;Lats1/2mut3;Nf1wt* (upper panel) and *H7;Lats1/2mut3;Nf1mut* (lower panel) mice. (**B**) Scatter plot of the total palpable tumor number in each mouse. *H7;Lats1^fl/+^;Lats2^fl/fl^;Nf1wt*, n=24. *H7;Lats1^fl/+^;Lats2^fl/fl^;Nf1mut*, *n* = 14. *H7;Lats1^fl/fl^;Lats2^fl/+^;Nf1wt*, *n* = 24. *H7;Lats1^fl/fl^;Lats2^fl/+^;Nf1mut*, *n* = 29. (**C**) Survival comparison between the *H7;Lats1^fl/+^;Lats2^fl/fl^;Nf1wt* and *H7;Lats1^fl/+^;Lats2^fl/fl^;Nf1mut* groups (left panel). Survival comparison between the *H7;Lats1^fl/fl^;Lats2^fl/+^;Nf1wt* and *H7;Lats1^fl/fl^;Lats2^fl/+^;Nf1mut* groups (right panel). *H7;Lats1^fl/+^;Lats2^fl/fl^;Nf1wt*, *n* = 24. *H7;Lats1^fl/+^;Lats2^fl/fl^;Nf1mut*, *n* = 14. *H7;Lats1^fl/fl^;Lats2^fl/+^;Nf1wt*, *n* = 24. *H7;Lats1^fl/fl^;Lats2^fl/+^;Nf1mut*, *n* = 29. (**D**) IHC of phospho-ERK1/2 and total ERK1/2 in *H7;Lats1/2mut3;Nf1wt* and *H7;Lats1/2mut3;Nf1mut* tumor sections. Scale bars: 50 μm. Two-tailed Student’s *t* test was applied to evaluate statistical significance in **B**. Log-rank statistical test was employed in **C**. Statistics are represented as the mean ± SEM. **P < 0.01.

We previously demonstrated that YAP/TAZ were activated in both human and mouse NF1-null cutaneous neurofibromas, a tumor driven by dysregulated RAS/MAPK signaling (27). Several studies have demonstrated that RAS/MAPK signaling may regulate the Hippo pathway through a WTIP-LATS interaction or Raf1-MST2 interaction (34, 35). Therefore, we hypothesized that the RAS/MAPK pathway may modify and accelerate schwannoma development. Since NF1 (neurofibromin) is a negative regulator of RAS and loss of NF1 activates RAS/MAPK signaling, to test our hypothesis, we crossed *H7;Lats1/2mut3* mice with *Nf1^fl/fl^* or *Nf1^fl/–^* (hereafter called *Nf1mut*) to obtain *H7;Lats1/2mut;Nf1mut* mice (Supplemental Figure 7A). As hypothesized, loss of *Nf1* significantly increased the tumor burden of *H7;Lats1/2mut3;Nf1mut* mice (Figure 6A). *H7;Lats1^fl/+^;Lats2^fl/fl^* and *H7;Lats1^fl/fl^;Lats2^fl/+^* mice developed about 9.2 and 21.2 tumors in each mouse, respectively (Figure 6B). However, *H7;Lats1^fl/+^;Lats2^fl/fl^;Nf1mut* and *H7;Lats1^fl/fl^;Lats2^fl/+^;Nf1mut* mice developed about 21.1 and 33.4 tumors in each mouse, respectively (Figure 6B).

Next, we investigated the impact of RAS/MAPK pathway activation via *Nf1* deletion on the overall survival of *H7;Lats1/2mut3* mice. At 100 days, approximately 93% of *H7;Lats1^fl/+^;Lats2^fl/fl^;Nf1mut* and 100% of *H7;Lats1^fl/fl^;Lats2^fl/+^;Nf1mut* had been sacrificed after reaching tumor size limits, whereas none of *H7;Lats1^fl/+^;Lats2^fl/fl^;Nf1wt* mice and only 24% of *H7;Lats1^fl/fl^;Lats2^fl/+^;Nf1wt* mice needed to be sacrificed (Figure 6C). Consistent with these findings, immunostaining confirmed that the loss of *Nf1* in *H7;Lats1/2mut3* tumor enhanced the expression of p-ERK, a RAS/MAPK pathway activation marker (Figure 6D and Supplemental Figure 7B), and these tumors were well circumscribed with hypercellular (Antoni A) areas and hypocellular (Antoni B) areas of predominantly organized Schwann cells, consistent with schwannomas histologically and molecularly (Supplemental Figure 8).

Taken together, these data suggest that the RAS/MAPK pathway may serve as a modifier of schwannomagenesis and that inhibition of RAS/MAPK signaling may serve as an alternative therapeutic strategy.

### MAPK pathway inhibition sensitizes schwannoma to JQ1 treatment.

**Figure 7:**
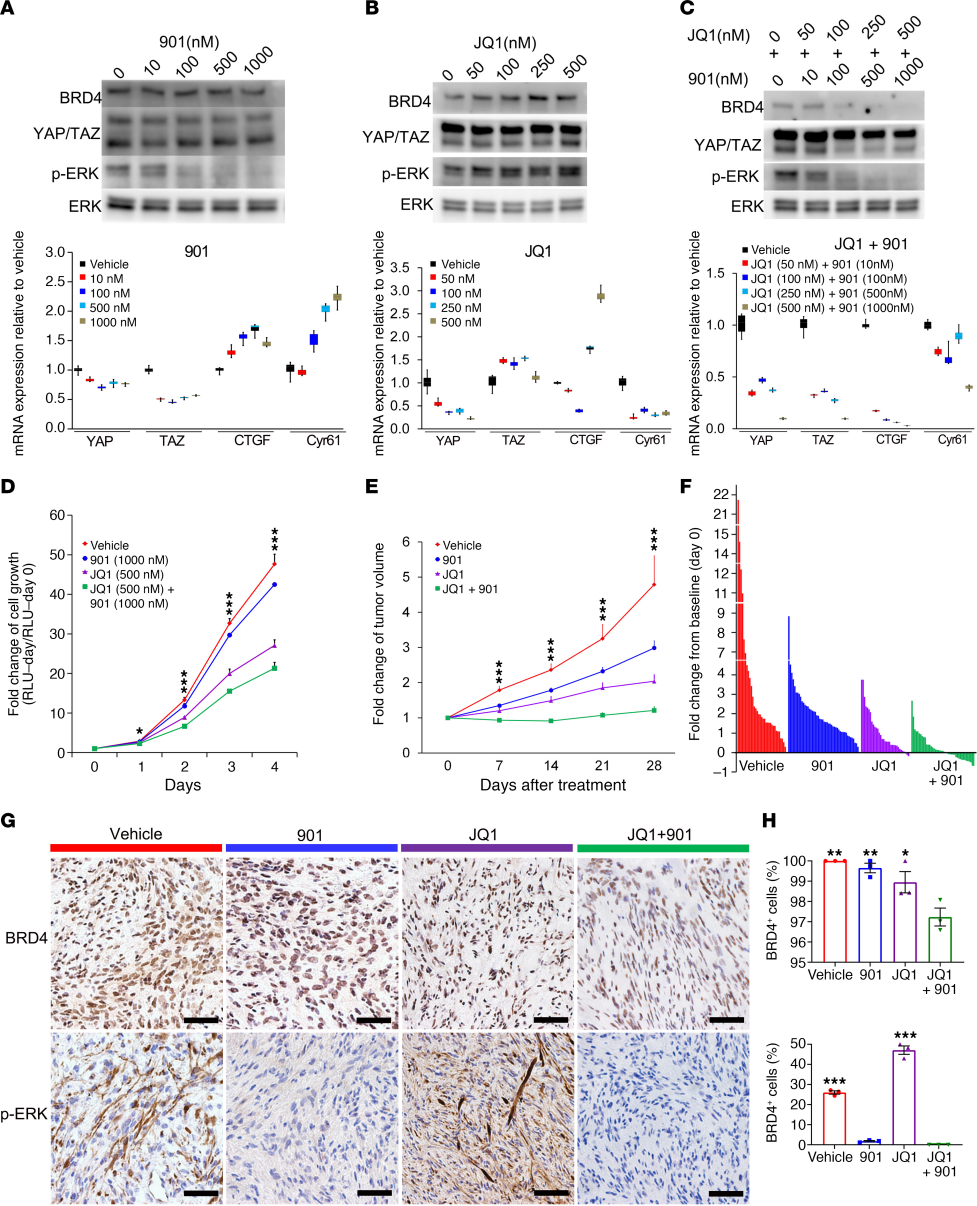
MAPK pathway inhibition sensitizes schwannoma to JQ1 treatment. (**A–C**) Western blot (top) and qPCR (bottom) analysis of a mouse schwannoma cell line (1162, generated from a schwannoma of a *H7;Lats1/2mut3* mouse) treated with 901 (**A**), JQ1 (**B**), or both (**C**). (**D**) Effect of treatment of 901, JQ1, or both on mouse schwannoma cell growth using ATP CellTiter Glo assay. *n* = 3/group. (**E**) Fold change of tumor volume with treatment of 901, JQ1, or both on tumor-burdened *H7;Lats1/2mut3* mice. Vehicle, *n* = 34. 901, *n* = 48. JQ1, *n* = 33. JQ1 + 901, *n* = 43. (**F**) Waterfall plot depicting fold change of tumor volume from baseline (day 0) after treatment with 901, JQ1, or both. Vehicle, *n* = 34. 901, *n* = 48. JQ1, *n* = 33. JQ1 + 901, *n* = 43. (**G**) IHC of BRD4 and phospho-ERK1/2 in mouse schwannomas treated with vehicle, 901, JQ1, or both. (**H**) Quantification of IHC in **G**. Scale bars: 50 μm. All statistical comparisons made to JQ1 + 901. *n* = 3/group. One-way ANOVA was applied to evaluate statistical significance in **D** and **E**. Box plots show median (line) and 25th to 75th percentile (box). The end of the whiskers represents the minimum and the maximum of all of the data in **A**, **B**, and **C**. One-way ANOVA with Tukey’s test for multiple comparisons were applied to evaluate statistical significance in **H**. All statistics are represented as the mean ± SEM. *P < 0.05, **P < 0.01, ***P < 0.001.

There are currently no targeted pharmacologic therapies for schwannomas. We sought to investigate the therapeutic effect of YAP/TAZ inhibition on schwannoma proliferation. Verteporfin was the first identified small molecule inhibitor of YAP and functions by disrupting the interaction between YAP and TEAD (36). It has been shown to have some degree of therapeutic effect on high YAP-expressing tumor mouse models (37, 38). Unfortunately, verteporfin does not disrupt the interaction between TAZ and TEAD, and an optimal inhibitory effect requires very high concentrations that make it unsuitable for in vivo use. Consistent with this, a previous report demonstrated that, although high-dose verteporfin alone suppressed *Lats1/2*-deficient tumor cell growth in vivo relative to vehicle, it increased risk of mortality due to toxicity (26). Additionally, a lower dose of verteporfin only showed a gradual and modest inhibition of tumor growth (26), indicating a need for alternative YAP/TAZ inhibitors. Recent studies suggest that pharmacologic inhibition of BRD4, a required cofactor for YAP/TAZ transcriptional activity, blunts growth of YAP/TAZ-addicted breast tumors. Interestingly, we also observed high BRD4 expression in YAP/TAZ-activated mouse schwannoma (Figure 7), indicating the therapeutic potential of BET inhibitors in the treatment of schwannoma (17). However, single inhibitor therapy of tumors often results in compensatory signaling upregulation and blunted therapeutic effects. For example, YAP/TAZ inactivation may cause compensatory lysosome-mediated activation of MAPK signaling in NF2 tumor growth (39). Additionally, YAP may also mediate resistance to MEK1/2 inhibition in neuroblastomas with hyperactivated RAS signaling (40). These studies suggest that targeting of both YAP/TAZ and MAPK signaling may provide additive or synergistic therapeutic benefit. Therefore, we tested the combined effect of a BRD4 inhibitor (JQ1) and MAPK pathway inhibitor (PD0325901, hereafter called 901) on schwannoma in vitro and in vivo. In mouse tumor cells, 901 substantially inhibited the phosphorylation of ERK1/2 and slightly inhibited the mRNA expression of YAP/TAZ but not their downstream target genes CTGF and Cyr61 (Figure 7A). JQ1 treatment alone downregulated mRNA expression of YAP and Cyr61, indicating partial inhibition of YAP/TAZ transcriptional activity (Figure 7B). Consistent with previous findings, we observed compensatory activation of the MAPK pathway upon JQ1 treatment (Figure 7B) (39). When we combined 901 and JQ1, we found dramatic inhibition of ERK1/2 phosphorylation and downregulation of YAP/TAZ transcriptional targets (Figure 7C). These data suggest a synergistic inhibitory effect of MAPK and Hippo pathway targeting that enables circumvention of established compensatory signaling mechanisms.

Next, we assessed whether tumor cells were sensitive to combined treatment of 901 and JQ1 in vitro. 901 treatment alone slightly suppressed the proliferation of tumor cells, while JQ1 alone demonstrated a greater effect. Combined 901 and JQ1 treatment significantly slowed schwannoma cell growth, outweighing the effect of either drug alone (Figure 7D). In order to validate our findings in vivo, we administered 901 and JQ1 in *H7;Lats1/2mut3* mice. These mice developed tumors around 2–3 months of age. Once the largest tumor diameter reached 5–10 mm, mice were randomized into 4 groups and treated with vehicle, 901, JQ1, or a combination of 901 and JQ1. Consistent with our in vitro data, *H7;Lats1/2mut3* mice treated with either 901 or JQ1 showed slowed tumor growth. However, there was no significant reduction in tumor size from baseline, with only 3 of 33 (9.1%) JQ1-treated tumors seeing size reduction. When mice were treated with combined 901 and JQ1, we found a marked reduction in tumor growth (Figure 7E). Furthermore, nearly half of the tumors (44.2%, 19 of 43) atrophied after 1 month (Figure 7F). IHC of 901- and JQ1-treated tumors confirmed reduced BRD4 expression and ERK1/2 phosphorylation (Figure 7, G and H). Altogether, our data demonstrate that cotargeting YAP/TAZ transcriptional activity and the MAPK signaling pathway may be a promising therapeutic strategy for schwannoma.

## Discussion

### Dysregulated Hippo-Lats1/2-YAP/TAZ pathway leads to schwannoma development.

The significance of canonical Hippo signaling in schwannoma pathogenesis has long been hypothesized, despite a lack of definitive evidence. In the present study, we provide multiple lines of evidence supporting the requirement of canonical Hippo signaling in schwannomagenesis. Firstly, complete loss of both *Lats1* and *Lats2* in the Hoxb7^+^ lineage resulted in multiple schwannoma formation. While the key effectors of the Hippo pathway, YAP/TAZ, are also regulated by alternative pathways such as Wnt and GPCR signaling (41, 42), these alternative modes of regulation still function through LATS1/2. In addition, activation of the Hippo pathway by reconstituting *NF2* expression in *NF2*-null breast cancer cell lines results in a robust LATS1/2-dependent inhibition of *YAP/TAZ* activity (43). Secondly, ablation of *YAP/TAZ* resulted in a gene dosage–dependent reduction in tumor burden and extension of life span. Although genetic ablation of *YAP/TAZ* only partially rescued the tumor phenotype of *H7;Lats1/2mut3* mice, YAP/TAZ expression was still detectable in these tumors, suggesting incomplete Cre recombination, resulting in positive selection of YAP- or TAZ-retaining tumor cells. This is supported by our single cell clonal analysis, which demonstrated retention of WT *TAZ* alleles in expanding clones only. This natural limitation of the *Cre-LoxP* system may be due to impaired chromatin accessibility (44). Lastly, although the functions of INI1/SMARCB1 and LZTR1 in schwannomagenesis are still unclear, recent research shows that subsequent biallelic loss of *Nf2* is necessary to induce schwannoma formation (45). Collectively, our data demonstrate that the dysregulation of Hippo-Lats1/2-YAP/TAZ signaling drives schwannomagensis.

### Hippo pathway dysregulation in schwannoma and cancer.

Loss of function of the core Hippo pathway kinases has been widely implicated in murine tumor models (9, 26, 46–48). However, with the exception of *NF2* mutations in human schwannoma (49-66%), very few Hippo pathway mutations have been found in human tumors. Only 2% and 1% of human schwannomas carry *Lats1* and *Lats2* mutations, respectively (49), and no *Mst1/2* mutation has been reported in human cancer. One possibility for this discrepancy is the functional redundancy of MST1 and MST2 or LATS1 and LATS2. Accordingly, we and others must ablate 4 alleles of *Lats1/2* in order to completely block the Hippo pathway for schwannoma induction, making human sporadic loss of function mutations unlikely. Another possibility is epigenetic silencing of these genes. Studies show that promoter methylation of *Lats1* and *Lats2* is common in schwannomas (17% and 30%, respectively) (49), while promoter methylation of *Mst1* and *Mst2* has been detected in 37% and 17% of sarcomas, respectively (50). These findings suggest that nonmutational mechanisms may also inactivate the Hippo pathway and play important roles in tumorigenesis.

It is worth noting that *YAP/TAZ* may be dispensable for tumorigenesis in certain cancers such as malignant hematopoiesis (51). However, our present work and other studies suggest that *YAP/TAZ* are likely required for tumors that form as a consequence of Hippo pathway mutations. If true, then the mutational status or promoter methylation of Hippo pathway components may be used to predict the therapeutic potential of *YAP/TAZ* inhibitors.

### YAP/TAZ and MAPK pathways act as the cotherapeutic target of schwannoma.

In addition to their essential roles in regulating tumor proliferation and stemness (52), *YAP/TAZ* also enable resistance to chemotherapy and targeted therapy (53). YAP/TAZ are also dispensable for tissue homeostasis (including of the Hoxb7^+^ Schwann cell lineage), making them ideal therapeutic targets (51, 52). Unfortunately, our single cell analyses clearly indicate that complete inhibition of both YAP and TAZ is required to block schwannoma cell proliferation. Therefore, adequate pharmacologic therapy necessitates targeting of both YAP and TAZ. However, there is currently no TAZ-specific inhibitor.

Alternatively, researchers had looked to transcriptional coactivators of YAP/TAZ for pharmacologic targets. BRD4 is one of the required transcriptional coactivators of YAP/TAZ. It has been shown that YAP/TAZ target genes have significant vulnerability to BRD4 inhibitors and that a relevant fraction of BRD4’s oncogenic functions are associated with YAP/TAZ (17). In line with these notions, a majority of the cell population in our mouse schwannoma tumor highly expressed BRD4 and YAP/TAZ. In addition, BRD4 inhibition by JQ1 partially blocked transcriptional activity of YAP/TAZ and reduced mouse schwannoma growth. However, when compared with the tumor size before JQ1 treatment, only 3 of 33 tumors atrophied, while the majority of tumors continued growing. These data suggest that YAP/TAZ inhibition by JQ1 results in cytostasis rather than cell death. Because our single cell clone forming assay also demonstrated that complete YAP/TAZ loss may only induce cytostasis, we looked for alternative pathways that may serve as adjunct targets. Many studies, including the present, indicate that BET inhibition activates compensatory MAPK signaling to provide a cell-protective effect (54). As we expected, combined pharmacologic targeting of BRD4 and the MAPK pathway achieved promising therapeutic results on mouse schwannoma. We found nearly half of the tumors atrophied, indicating that cotreatment may trigger tumor apoptosis or cell death. Furthermore, combined treatment decreased the phosphorylation of ERK and protein levels of BRD4 and YAP/TAZ, suggesting a synergistic effect. Interestingly, a previously published study demonstrated that cotargeting of BRD4 and MAPK signaling promoted cell death in a NF1 loss-of-function mouse model of malignant peripheral nerve sheath tumor (MPNST) (55). The authors demonstrated that targeting of both pathways resulted in PRC2/SUZ12-mediated epigenetic remodeling that repressed RAS transcriptional output. Given the crosstalk between the Hippo and MAPK pathways, it is interesting to postulate that JQ1 sensitivity in MPNST may be due to a YAP/TAZ-dependent mechanism. It is also known that phosphorylation of ERK induces cyclin D expression, while YAP/TAZ activation upregulates cyclin dependent kinase 6 (CDK6) expression (56, 57). Cyclin D forms a complex with and activates CDK4/6, an important regulator of DUB3-mediated deubiquitination and stability of BRD4 (58, 59). This may represent another mechanism through which cotargeting BRD4 and phosphorylation of ERK results in decreased protein level of BRD4 and impairment of schwannoma proliferation.

Finally, our studies using *Nf1* deletion to activate the MAPK pathway suggested that this signaling pathway might modify schwannoma development. However, neurofibromin also regulates a number of MAPK-independent pathways; thus, it is possible that one or more of these other pathways may also affects schwannoma development.

In conclusion, we provide genetic evidence that dysregulation of Hippo-Lats1/2-YAP/TAZ signaling mediates schwannomagenesis. Furthermore, the MAPK pathway acts as a modifier of schwannoma development, and cotargeting of YAP/TAZ and the MAPK pathway is a promising therapeutic strategy for schwannomas. Although the herein described mouse model produces schwannomas with matching histology, the spectrum of this mouse tumor syndrome is beyond the scope of human schwannomas or schwannomatosis. Nonetheless, our model provides a framework to begin to dissect the molecular mechanisms of schwannomagenesis and identify novel therapeutic targets.

## Methods

Mice. The *Nf1*-KO (60), *Nf1* flox (61), *Hoxb7-Cre* (62), *Lats1* flox (63), *Lats2* flox (63), *Yap* flox (64), *Taz* flox (65), and athymic nude mice (*Foxn1^–/–^*) were purchased from Jackson Laboratory. Genotyping was performed by PCR as previously reported (60–65). All mice were housed in the Animal Care Facility at the UT Southwestern Medical Center (Dallas, Texas, USA).

### Animal studies.

Mice were monitored and sacrificed when the largest tumor diameter reached 1 cm. The hair of mice was removed, and total palpable tumor number was counted. The dates of sacrifice were documented for survival analysis. Tumors arising near the spine could occasionally infiltrate and compress the spinal cord, leading to paralysis of mice. These mice were euthanized before the largest tumor diameter reached 1 cm. In this case, the dates of sacrifice were still documented; however, the total tumor number was excluded for quality purposes.

To analyze the therapeutic effect of JQ1 (Cayman Chemical), 901 (Selleck Chemicals), or combined JQ1 and 901 treatment on tumor-burdened *H7;Lats1/2mut3* mice, we closely monitored the tumor size of each mouse. When the biggest tumor diameter reached 0.5–1 cm, the tumor-burdened *H7;Lats1/2mut3* mice were randomly assigned into 4 groups (5 mice/group). Group 1 received vehicle daily. Group 2 received JQ1 (45 mg/kg i.p.) daily in a 10% (2-hydroxypropyl)-β-cyclodextrin (MilliporeSigma) solution. Group 3 received 901 by oral gavage daily at 1.5 mg/kg (vehicle [0.5% (w/v) methylcellulose (MilliporeSigma) solution with 0.2% (v/v) Tween 80 (MilliporeSigma)]). Group 4 was administered a combination of JQ1 (45 mg/kg i.p.) and 901 (1.5 mg/kg, oral gavage) sequentially (55). All groups were treated for 1 month. Tumor size was measured weekly, and tumor volume was calculated by measuring length and width of the lesion with the formula length × width^2^ × 0.52 (66).

### E13.5 DRG/nerve roots and tumorsphere cell culture.

E13.5 DRGs/nerve roots were isolated, and neurospheres were generated as previously described (29). Briefly, mouse embryos were removed from anesthetized 13.5-day-old pregnant female mice. Embryos were sacrificed, and the spinal cord of each embryo was removed. DRGs/nerve roots were dissected from the vertebral column with the aid of a stereomicroscope. The DRGs/nerve roots were digested with 1 mg/mL collagenase (Thermo Fisher Scientific) at 37°C for 30 minutes and then washed twice with DMEM/F12 (Thermo Fisher Scientific). The cells were counted and plated on uncoated, ultra-low attachment 6-well plates (Corning) to allow the sphere formation in proliferation media: DMEM/F12 containing penicillin/streptomycin (RPI Research Product; 0.1%); fungizone (Thermo Fisher Scientific, 40 mg/mL); B27 (Thermo Fisher Scientific, without vitamin A), epidermal growth factor (Thermo Fisher Scientific, 20 ng/mL), and basic fibroblast growth factor (40 ng/mL) (Sigma-Aldrich). The sphere cells were fed every 3–4 days and passaged every 7 days. Sphere cells were then seeded on fibronectin-coated cell culture plates for monolayer culture with aforementioned proliferation media.

For tumorsphere cell culture, the tumors were removed from the mice. Tumors were minced with fine scissors, and tumorsphere cell culture was performed using the same procedure mentioned above. The schwannoma cell line 1162 was generated in this way from a mouse schwannoma tumor harvested from a *Hoxb7-Cre;Lats1/2mut3* mouse.

### In vitro growth assays.

ATP CellTiter Glo assay (Promega) was performed per manufacturer’s instructions. Luminescence was quantified via Synergy HT 96-well plate reader (BioTek).

### Cytoplasmic and nuclear fractionation.

Cytoplasm and nuclear extracts were isolated as previously described (67), with some modifications. Cells were harvested by trypsin-EDTA (MilliporeSigma), collected by centrifugation (5000*g*, 4°C, 5 minutes), and washed twice in ice-cold PBS. The cell pellets were resuspended in 1 volume PBS and 2 volume hypotonic lysis buffer (HLB; 10 mM Tris-HCl, pH 7.4, 10 mM NaCl, 3 mM MgCl_2_, and protease inhibitors [MilliporeSigma]) and incubated for 10 minutes on ice. NP-40 (MilliporeSigma) was added at a final concentration of 0.1% total volume. The lysed cell solution was placed on ice for 10 minutes. Nuclei were then pelleted by centrifugation at 5000*g* for 5 minutes at 4°C, and supernatant containing cytoplasmic proteins was collected and stored at −80°C. The nuclear pellet was washed twice with ice cold 0.1% NP40-HLB buffer. The nuclear pellet was resuspended in 1 volume of 0.1% NP40-HLB buffer with protease inhibitors, and then 0.2 volume of 5× nuclei lysis buffer (0.5% NP40, 500 mM NaCl, and 50 mM Tris; MilliporeSigma) was added. The lysed nuclei solution was incubated on ice for 10 minutes and then centrifuged at 21,000*g* at 4°C for 10 minutes. The supernatant containing nucleus proteins was collected and stored at −80°C. Both cytoplasmic and nuclear fractions were analyzed by Western blotting.

### RNA Isolation, cDNA Synthesis, quantitative PCR.

RNEasy mini kit (Qiagen) was used to isolate total RNA from cells, followed by cDNA synthesis with iScript Select cDNA synthesis kit (Bio-Rad) and quantitative PCR (qPCR) using iTaq Universal SYBR Green Supermix (Bio-Rad). The following primers were used: YAP forward (YAP-F): 5′-ACCAATAGTTCCGATCCCTTTC-3′; YAP reverse (YAP-R): 5′-TGTCTCCTGTATCCATTTCATCC-3′; TAZ-F: 5′-CGTCCATCACTTCCACCTC-3′; TAZ-R: 5′-GTTGGTTCTGAGTCGGGTG-3′; β-actin-F: 5′-TTCTACAATGAGCTGCGTGTG-3′; β-actin-R: 5′-GGGGTGTTGAAGGTCTCAAA-3′; CTGF-F: 5′-CTGCCTACCGACTGGAAGAC-3′; CTGF-R: 5′-CATTGGTAACTCGGGTGGAG-3′; Cyr61-F: 5′-GCTCAGTCAGAAGGCAGACC-3′; Cyr61-R: 5′-GTTCTTGGGGACACAGAGGA-3′;

### IHC.

For IHC, paraffin sections were deparaffinized, rehydrated, and subjected to antigen retrieval prior to incubation with the primary antibodies as described below. The primary antibodies were visualized by treating the sections with biotinylated secondary antibody followed by amplification with peroxidase-conjugated avidin and DAB substrate per manufacturer’s protocol (Vector Laboratories). The primary antibodies used in this study were as follows: rabbit anti–S100 β (Agilent, catalog Z0311, RRID:AB_10013383), rabbit anti-GFAP (Agilent, catalog Z0334, RRID:AB_10013382), rabbit anti-SOX10 (Abcam, catalog ab180862, RRID:AB_2721184), rabbit anti–Collagen IV (Abcam, catalog ab6586, RRID:AB_305584), rabbit anti-YAP (Cell Signaling Technology, catalog 14074, RRID:AB_2650491), rabbit anti-TAZ (ABclonal, catalog A8202, RRID:AB_2721146), rabbit anti–p-H3 (Cell Signaling Technology, catalog 9701, RRID:AB_331535), rabbit anti-LATS1 (Proteintech, catalog 17049-1-AP, RRID:AB_2281011), rabbit anti-LATS2 (Proteintech, catalog 20276-1-AP, RRID:AB_10697657), rabbit anti–total ERK1/2 (Cell Signaling Technology, catalog 4695, RRID:AB_390779), rabbit anti–p-ERK1/2 (Cell Signaling Technology, catalog 4370, RRID:AB_2315112), and rabbit anti-NF2 (Thermo Fisher Scientific, catalog PA5-82060, RRID:AB_2789221).

### Mouse whole spinal cord dissection.

Whole spinal cord dissection was performed as previously reported (68). To perform mouse anesthesia, a mixture of ketamine (10 mg/mL) and xylazine (1 mg/mL) solution 9 (provided by UT Southwestern ARC) (100 μL per 25g of mouse weight) was administered i.p. After 15–20 minutes, mice were placed face-up in a surgical field, and the chest area was sprayed with 70% ethanol (Pharmco). The left thoracic cage was removed, a catheter was installed in the heart left ventricle, and mice were perfused intracardially with 4% paraformaldehyde (MilliporeSigma). Then, the mouse was prepared for microscopic dissection by removing gross tissue (cervical decapitation, whole skin removal, and all internal organs). Next, muscle and other tissue were carefully removed, and bones from the vertebrate column were broken one by one under dissection microscope to end up with intact spinal cord and peripheral nerves. Finally, whole spinal cord and peripheral nerves were rinsed with PBS 1× (Thermo Fisher Scientific) and immersed in 10% formalin-buffered solution (MilliporeSigma).

### DRG volume measurement.

DRG and spinal nerves were dissected out under a microscope after being fixed and decalcified in 5% formic acid (MilliporeSigma). The tumor volume was calculated according to a specific formula as volume = length × width^2^ × 0.52, which approximates the volume of a spheroid.

### Transplantation experiments.

SN implantation of E13.5 DNSCs was performed as previously reported (69). Briefly, mice were anesthetized by i.p. injection of 120 μL of a mixture of ketamine (10 mg/mL) and xylazine (1 mg/mL) solution (provided by UT Southwestern ARC). A skin incision was made above the femur. Using iris scissors, a pocket was created within the quadriceps muscles to expose the SN. A total of 40 μL of L15 medium (Thermo Fisher Scientific) containing 1 ×10^6^ viable DNSCs was then deposited into this pocket so that DNSCs could be in contact with the SN. The quadriceps muscles were then closed with 4-0 Vicryl suture (Ethicon), and the skin was closed with 5-0 prolene suture (Ethicon).

For s.c. implantation, the nude mouse was manually restrained by hand and then placed on a clean towel. A total of 1 × 10^6^ DNSCs or tumor cells was injected under the skin of the shoulder area.

### Genotyping PCR and single cell nested PCR.

Genotyping PCR primers are as follows: *Lats1*-F: 5′-CCTTTATGCTGATGCCCTAAGA-3′; *Lats1*-R: 5′-ATGAATGAACCTGAGGCTGC-3′; *Lats2*-F: 5′-AAAGCACAGGGCCTTTTACA-3′; *Lats2*-R: 5′-ACACATTCCCCTCCACTGAC-3′; *β-actin*–F: 5′-CCTAGGCACCAGGGTGTGAT-3′; *β-actin*–R: 5′-TCACGGTTGGCCTTAGGGTT-3′.

For single cell nested PCR, single cells were isolated by flow cytometry into each well of a 96-well PCR plate. Single cell nested PCR was performed as previously reported (70). Briefly, 10 μL of single cell lysis buffer was added to well containing 1 single cell/well. A total of 90 μL of reaction mixture including first PCR primers was added. PCRs were performed in 100 μL of reaction buffer containing 10 mM Tris-HCl (pH 8.3), 50 mM KCl, 2.0 mM Mg^2+^, 200 μM of each dNTP, and 1.25 units Taq polymerase. A total of 25 pmol of each primer was used. The primers were designed as follows: the outer forward primer (1F) and reverse primer (1R) sequences were located upstream and downstream of the 2 *loxP* sites of *TAZ*, respectively; The inner forward primer (2F) and reverse primer (2R) sequences were located inside the 1F and 1R sequence but outside the 2 *loxP* sites of *TAZ* such that the flox and Δfloxed alleles of *TAZ* could be detected (Figure 5). Two reverse primers with recognition sequences within the loxP-flanked exon were also generated (R1.5 and R2.5). The primer sequences are as follows: *TAZ*-F1: 5′-ATCTTGCCTCTGGAGCACT-3′; *TAZ* R1: 5′-GGCAAAGCACAGGGTAAGAA-3′; *TAZ* R1.5: 5′-TCCTTTCTGGAAAGTTGCATT-3′; *TAZ* F2: 5′-GGCCACTGCATTTGACATTC-3′; *TAZ* R2: 5′-CATCATCAGAAAACAGCAGCA-3′; *TAZ* R2.5: 5′-AATGCTTCTCCCAAGACTG-3′.

Two consecutive PCRs with nested primers were performed. For the first PCR with outer primers (F1 + R1 + R1.5), following 5 minutes of incubation at 94°C, 30 cycles were carried out with denaturing for 30 seconds at 94°C, annealing for 30 seconds at 60°C, and extension for 2 minutes at 72°C, with a final extension step of 10 minutes at 72°C. For the second PCR with inner primers (F2 + R2 + R2.5), 20 μL of the reaction buffer described above and 1 μL of the first PCR product were mixed. Thirty cycles of 35 seconds at 94°C, 30 seconds at 70°C, and 40 seconds at 72°C were followed by a final extension step of 10 minutes at 72°C.

### Western blot analysis.

Protein isolation and subsequent Western blot analysis was performed as described previously (66). The following antibodies were used: rabbit anti-YAP/TAZ (Cell Signaling Technology, catalog 8418, RRID:AB_10950494), rabbit anti–Histone H3 (Cell Signaling Technology, catalog 9717, RRID:AB_331222), mouse anti-GAPDH (Santa Cruz Biotechnology Inc., catalog sc-47724, RRID:AB_627678), rabbit anti-BRD4 (Bethyl, catalog A700-004, RRID:AB_2631885), rabbit anti-ERK1/2 (Cell Signaling Technology, catalog 4695, RRID:AB_390779), rabbit anti–p-ERK1/2 (Cell Signaling Technology, catalog 4370, RRID:AB_2315112), rabbit anti-LATS1 (Proteintech, catalog 17049-1-AP, RRID:AB_2281011), rabbit anti-LATS2 (Proteintech, catalog 20276-1-AP, RRID:AB_10697657), rabbit anti-MST1 (Cell Signaling Technology, catalog 3682, RRID:AB_2144632), rabbit anti-MST2 (Cell Signaling Technology, catalog 3952, RRID:AB_2196471), rabbit anti-NF2 (Thermo Fisher Scientific, catalog PA5-82060, RRID:AB_2789221), rabbit anti-SAV1 (Cell Signaling Technology, catalog 13301, RRID:AB_2798176), and rabbit anti-MOB1 (Cell Signaling Technology, catalog 13730, RRID:AB_2783010).

### shRNA and lentiviral constructs.

The pLKO.1-TRC cloning vector was a gift from David Root (Addgene plasmid no. 10878; http://n2t.net/addgene:10878; RRID:Addgene_10878) (71). pLKO-shRNA plasmids were generated as described on the website: https://www.addgene.org/tools/protocols/plko/ The following constructs were used: pLKO-shYAP-5: 5′-TGAGAACAATGACAACCAATA-3′ (TRCN0000238436); pLKO-shYAP-6: 5′-GCAGACAGATTCCTTTGTTAA-3′ (TRCN0000095864); pLKO-shYAP-7: 5′-GAAGCGCTGAGTTCCGAAATC-3′ (TRCN0000238432); pLKO-shYAP-8: 5′-TCCAACCAGCAGCAGCAAATA-3′ (TRCN0000238433); pLKO-shTAZ-6: 5′-CAGCCGAATCTCGCAATGAAT-3′ (TRCN0000095951); pLKO-shTAZ-7: 5′-CCATGAGCACAGATATGAGAT-3′ (TRCN0000095953); pLKO-shTAZ-8: 5′-GTGATGAATCAGCCTCTGAAT-3′ (TRCN0000095952); pLKO-shTAZ-9: 5′-CCTTCTTTAAGGAGCCCGATT-3′ (TRCN0000095950). Packaging vectors psPAX2 and pMD2.g were gifts from Didier Trono (Addgene plasmid no. 12260, http://n2t.net/addgene:12260; RRID:Addgene_12260; Addgene plasmid no. 12259, http://n2t.net/addgene:12259; RRID:Addgene_12259). psPAX2 and pMD2.g packaging vectors were used for lentivirus production.

### Statistics.

All data are displayed as the mean ± SEM unless specified otherwise. For mouse survival analysis, Kaplan-Meier estimator with log-rank statistical test was employed. One-way ANOVA and 2-tailed Student’s *t* test and 1-way ANOVA with Tukey’s test for multiple comparisons were applied to evaluate statistical significance (*P < 0.05; **P < 0.01; ***P < 0.001).

### Study approval.

All mouse procedures were approved by IACUC at UT Southwestern Medical Center and conformed to NIH guidelines.

## Author contributions

Conceptualization and methodology, data analysis, writing, review and editing, and project management were contributed by ZC, DWC, and LQL. Investigations and data acquisition were contributed by ZC, JM, TS, YW, EH, YH, JPB, TJC, and LQL. Generation of mouse reagents was contributed by TJC. Writing and editing of the original draft were contributed by ZC, SL, and LQL. Visualization and data presentation were contributed by ZC, SL, and LQL. Supervision and funding acquisition were contributed by LQL.

## Supplementary Material

supplemental data

## Version 1 09/22/2020

In-Press Preview

## Version 2 10/15/2020

Electronic publication
