# Supplementary material for: Schwannoma development is mediated by Hippo pathway dysregulation and modified by RAS/MAPK signaling
Source: JCI Insight. 2020 Oct 15;5(20):e141514. doi: 10.1172/jci.insight.141514 (PMC7605536; doi:10.1172/jci.insight.141514)
Supplement: supplemental data [file jciinsight-5-141514-s220.pdf]

Supplemental Figures and Tables for:

Schwannoma Development is Mediated by Hippo Pathway Dysregulation and Modified by RAS/MAPK Signaling

Zhiguo Chen[1], Stephen Li[1,2], Juan Mo[1], Eric Hawley[3], Yong Wang[1], Yongzheng He[3], Jean-Philippe Brosseau[1,*], Tracey Shipman[1], D. Wade Clapp[3], Thomas J. Carroll[4,6], Lu Q. Le[1,5,6]

[1]Department of Dermatology, UT Southwestern Medical Center, Dallas, TX, USA
[2]Medical Scientist Training Program, UT Southwestern Medical Center, Dallas, TX, USA
[3]Department of Pediatrics, Indiana University School of Medicine, Indianapolis, IN, USA
[4] Department of Molecular Biology, UT Southwestern Medical Center, Dallas, TX, USA
[5]Comprehensive Neurofibromatosis Clinic, UT Southwestern Medical Center, Dallas, TX, USA
[6]Simmons Comprehensive Cancer Center, UT Southwestern Medical Center, Dallas, TX, USA
[*]Current address: Department of Biochemistry and Functional Genomics, University of Sherbrooke, Sherbrooke, Canada, J1E 4K8

Author for correspondence:
Lu Q. Le, M.D., Ph.D.
Professor
Department of Dermatology
Simmons Comprehensive Cancer Center
Hamon Center for Regenerative Science and Medicine
UT Southwestern Medical Center
Phone: (214) 648-5781
Fax: (214) 648-5553
E-mail: lu.le@utsouthwestern.edu

Conflict of interest:
The authors have declared that no conflict of interest exists.

Running title: Hippo Pathway in Schwannoma Development

Keywords: Hippo Pathway, Neurofibromatosis type 2 (NF2), Schwannoma, Schwannomatosis, Merlin, MAPK pathway, Schwann cell

Supplemental Figure 1

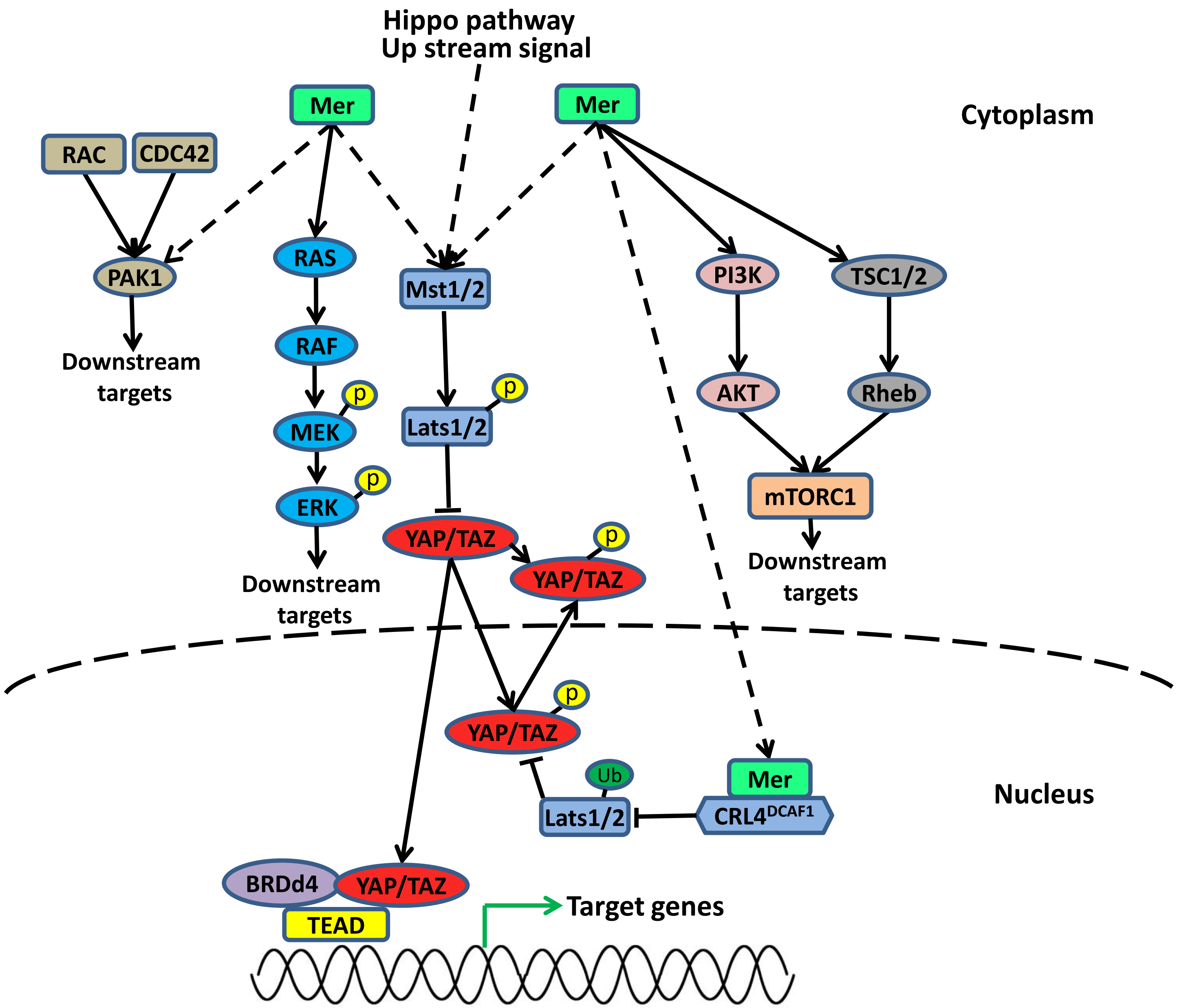

Supplemental Figure 1. Schematic diagram of Merlin and Hippo pathway signaling. Core Hippo pathway components are shown. Merlin (MER) activates the Hippo pathway by forming a complex with STE20-like protein kinase 1/2 (MST1/2; mammalian ortholog of Hippo). When phosphorylated and activated by upstream signals, Mst1/2 directly phosphorylates and activates large tumor suppressor homolog 1/2 (LATS1/2). In parallel, Merlin can recruits LATS1/2 kinases to the plasma membrane for phosphorylation and activation by MST1/2 kinases. Merlin can also modulate LATS1/2 activity through the cullin4 (CUL4)-RING E3 ubiquitin ligase complex (CRL4-DCAF1). Activated LATS1/2 in turn phosphorylates and induces cytoplasmic retention and degradation of the transcription factors Yes-associated protein or transcriptional coactivator with PDZ-binding motif (YAP/TAZ). When Hippo signaling is in the "off" state, YAP/TAZ are devoid of serine phosphorylation and so escape from degradation and cytoplasmic retention. They translocate into the nucleus, where they interact with transcriptional enhancer associated domain (TEAD) and other transcription factors such as bromodomain-containing protein 4 (BRD4) to co-activate the expression of target genes. Merlin also interacts with other signaling pathways such as the MAP kinase pathway and the PI3 kinase pathway to activate gene expression.

Supplemental Figure 2

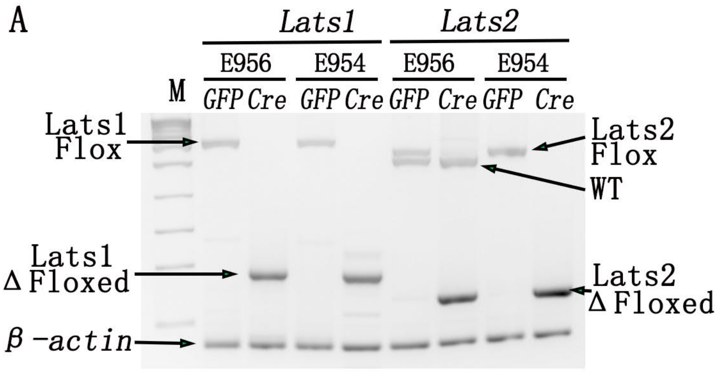

Supplemental Figure 2. *Lats1* or *Lats2* loss of heterozygosity in *H7;Lats1/2mut3* mice is required for schwannoma development.

(A) Genotyping analysis of *Lats1/2mut3* and *Lats1/2mut4* DNSCs with *Ad-GFP* or *Ad-Cre*. (B) Western blot analysis of LATS1 and LATS2 expression in indicated cells. E956 genotype: $\textit{Lats1}^{f/f}$;$\textit{Lats2}^{f/+}$; E954 genotype: $\textit{Lats1}^{f/f}$;$\textit{Lats2}^{f/f}$. *GFP*: *Ad-GFP. Cre*: *Ad-Cre*. M: molecular weight marker.

Supplemental Figure 3

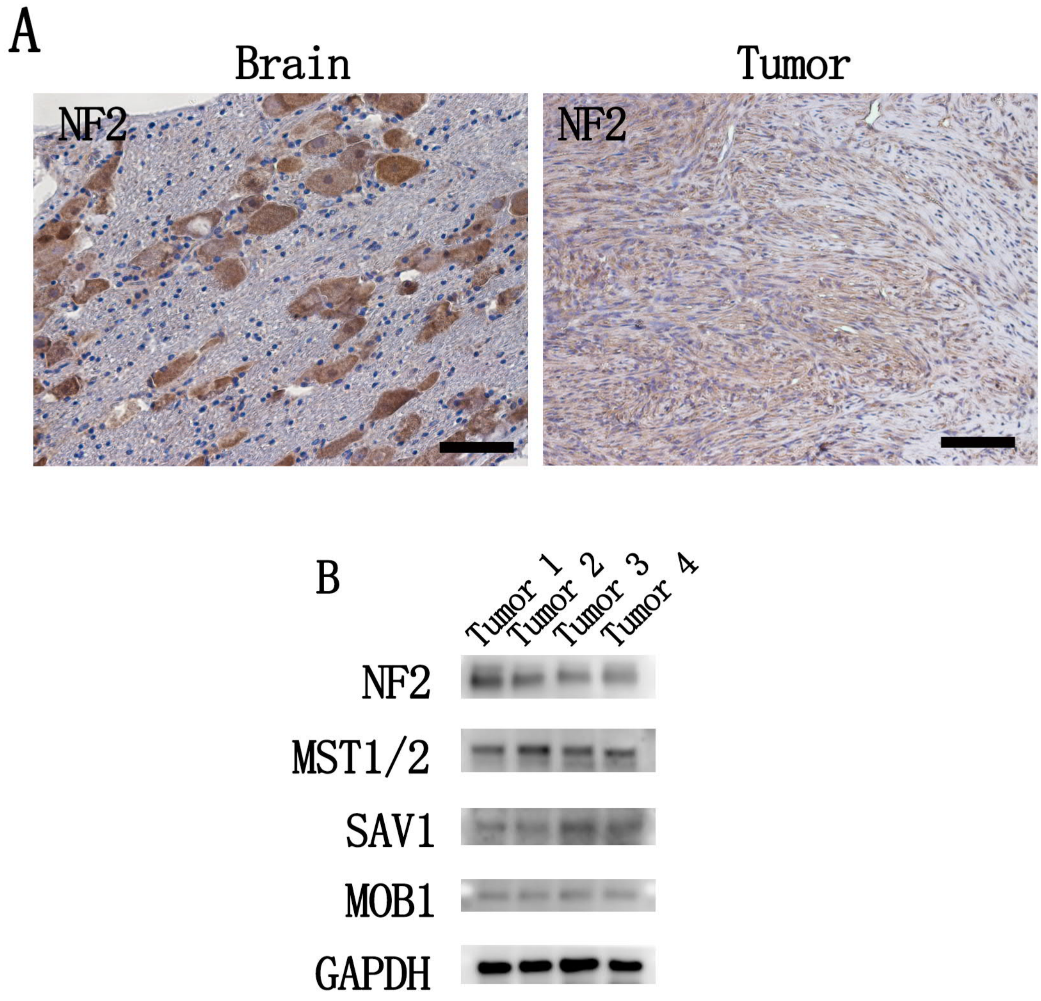

Supplemental Figure 3. NF2 and other Hippo pathway component expression in mouse schwannoma.

(A) Immunohistochemistry of NF2 on Brain (positive control) and tumor sections. (B) Western blot analysis of Hippo pathway components upstream of LATS1/2 on tumor tissues. Scale bars, 50 μm.

Supplemental Figure 4

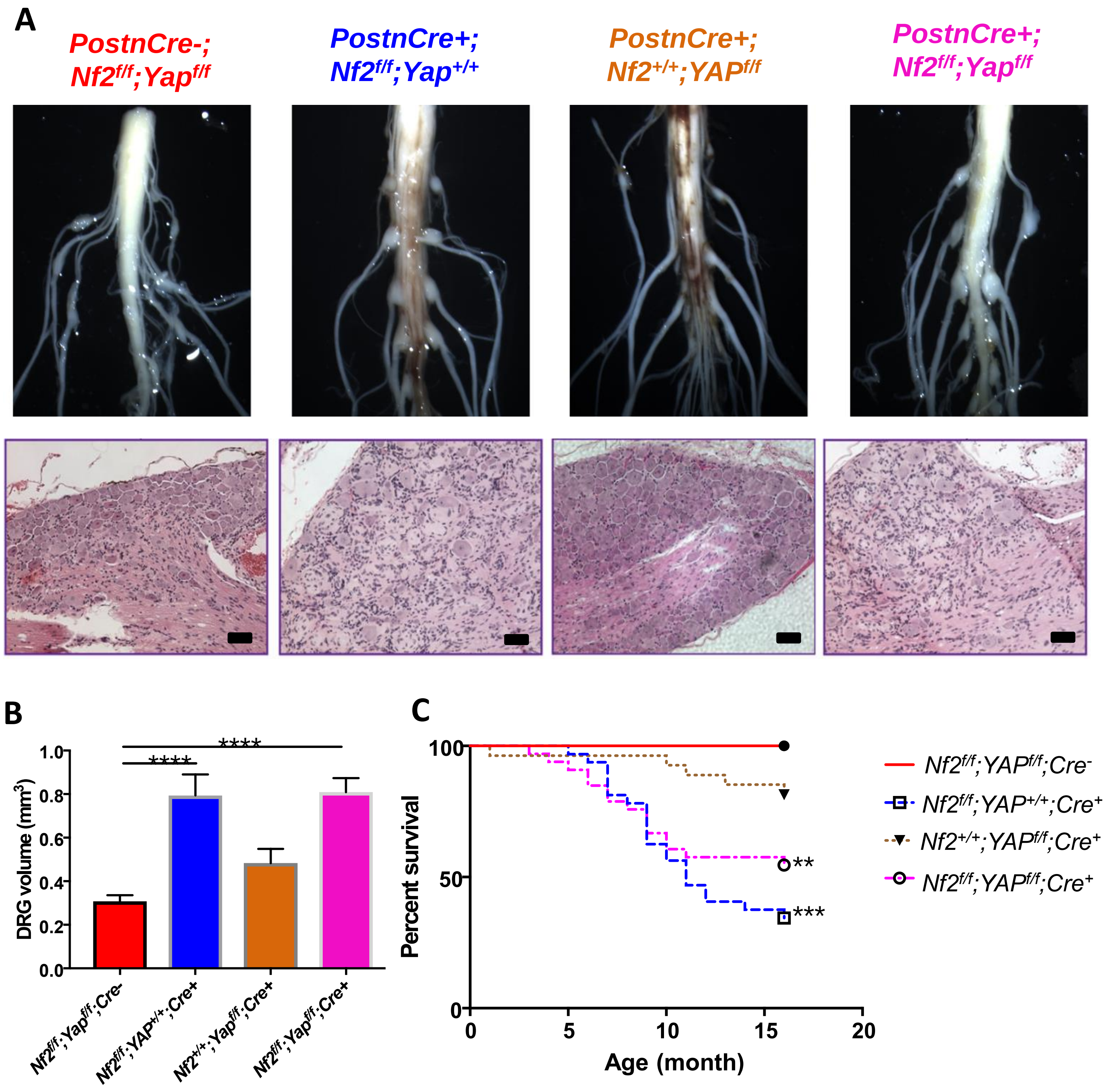

Supplemental Figure 4. YAP deletion did not affect tumor burden or lifespan.

(A) Representative pictures of spinal cord and peripheral nerve (upper panel) of depicted genotype; Histological evaluation of DRGs (Lower panel). (B) Average DRG volume of mice with different genotypes. (C) Kaplan-Meier plot illustrating the survival curve of mice with indicated genotypes. Scale bars, 50 μm.

Supplemental Figure 5

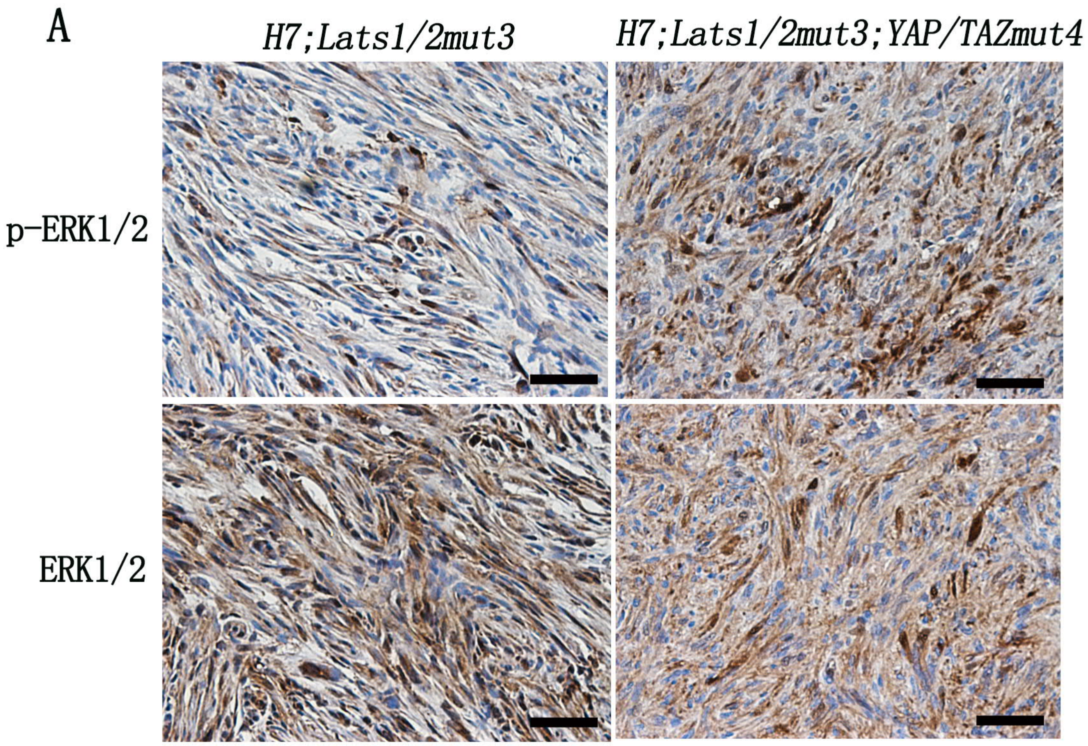

Supplemental Figure 5. Deletion of YAP/TAZ activates MAPK signaling.

(A) Immunohistochemistry of phospho-ERK1/2 and total ERK1/2 in *H7;Lats1/2mut3* and *H7;Lats1/2mut3;YAP/TAZ mut4* tumor sections. Scale bars, 50 μm.

Supplemental Figure 6

A

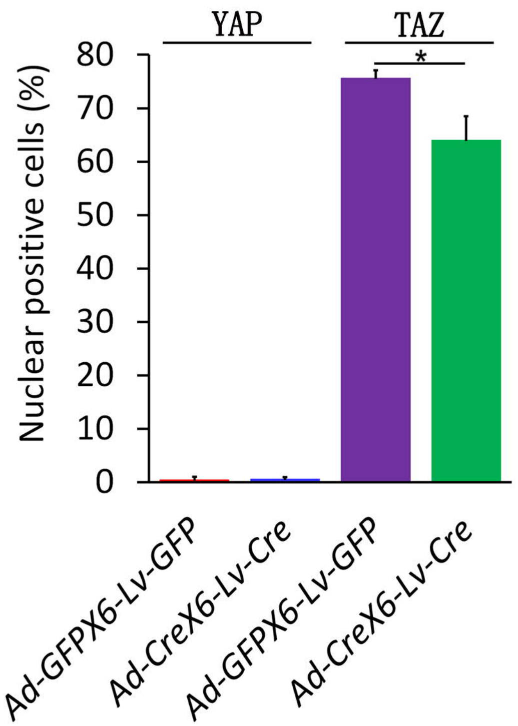

B

459T Cell line

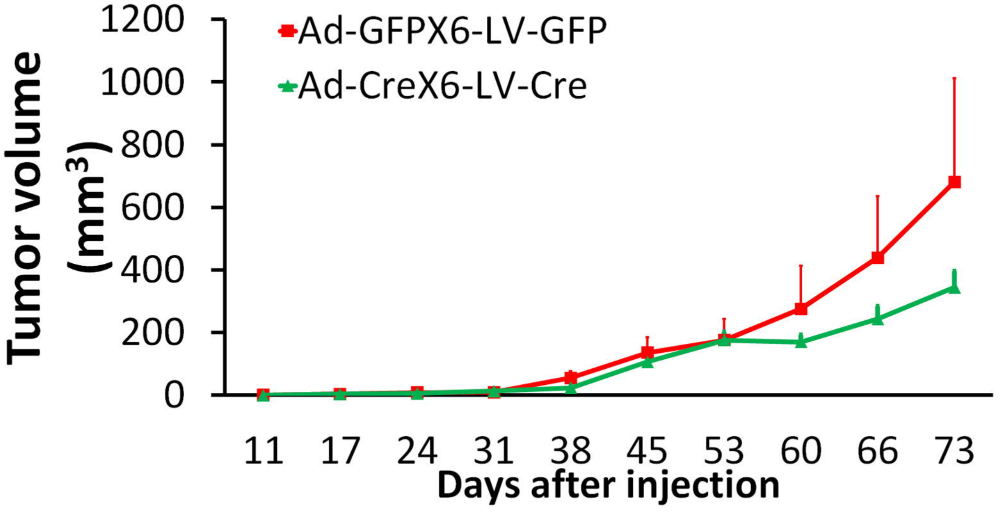

C

459T Cell line

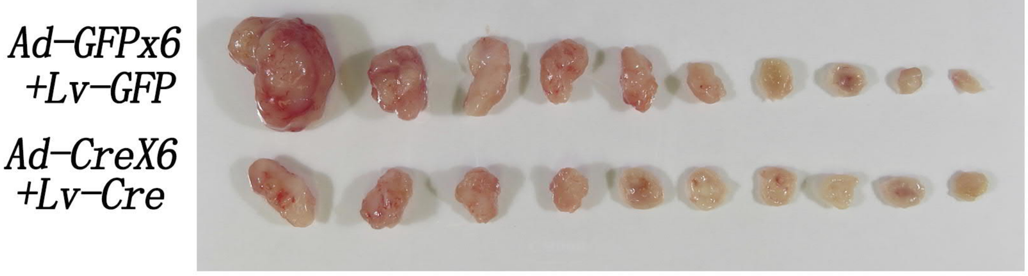

Supplemental Figure 6. Canonical Hippo signaling through YAP/TAZ is required for schwannomagenesis.

(A) Quantification of YAP and TAZ immunohistochemistry in Figure 5B. n = 5/group. (B) Tumor volume of 459T-*Ad-GFPX6-Lv-GFP* and 459T-*Ad-CreX6-Lv-Cre* schwannoma tumor in nude mice. N = 10/group. (C) Gross picture of tumors from experimental endpoint in (B). n = 10/group.

Supplemental Figure 7

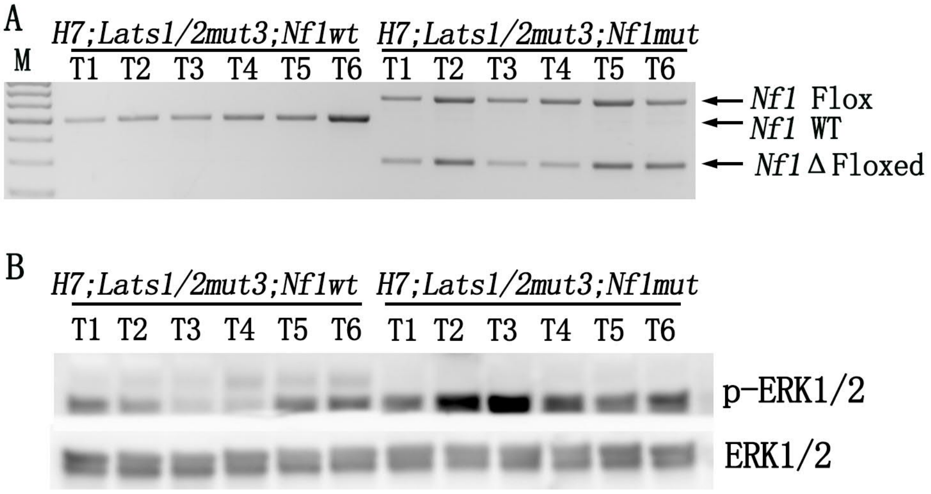

Supplemental Figure 7. MAPK pathway activation following NF1 loss.

(A) Genotyping of *Nf1* and (B) Western blot analysis of phospho-ERK1/2 and ERK1/2 in tumors from *Hoxb7-Cre;Lats1/2mut3;Nf1wt* and Hoxb7-*Cre;Lats1/2mut3;Nf1mut* mice.

Supplemental Figure 8

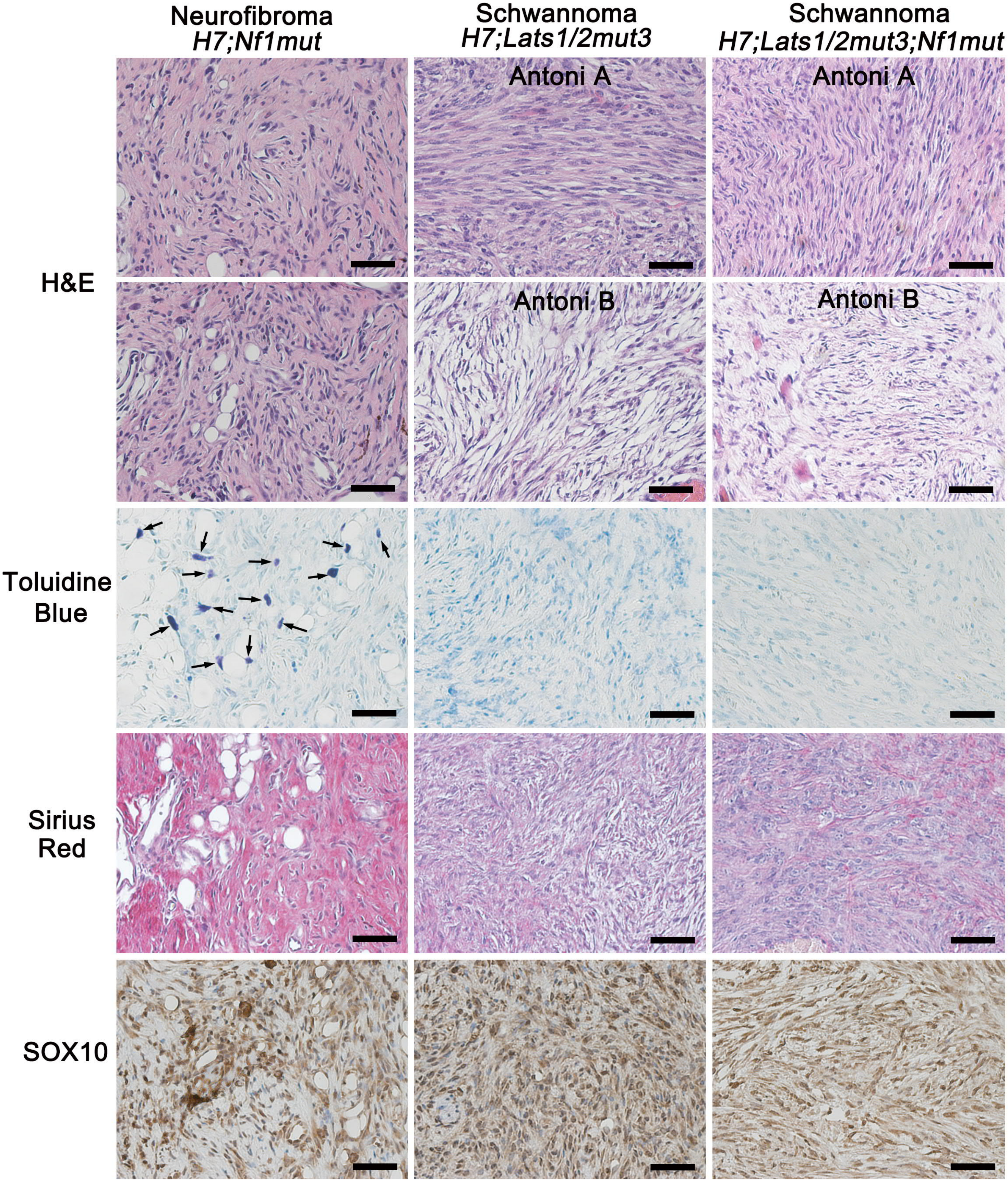

Supplemental Figure 8. *Hoxb7-Cre;Lats1/2mut3;Nf1mut* mice developed schwannoma and not neurofibroma.

Histological and molecular analysis by H&E staining, Toluidine blue staining (Mast cell marker, arrow), Sirius red staining (collagen marker) and SOX10 immunohistochemistry (neural crest lineage marker) of cutaneous neurofibroma from *Hoxb7-Cre;Nf1mut* mice (left column); schwannoma from *Hoxb7-Cre;Lats1/2mut3* mice (middle column) and schwannoma from *Hoxb7-Cre;Lats1/2mut3;Nf1mut* mice (right column). Scale bars, 50 µm.

Supplemental Table 1. Genotype and phenotype of *H7;Lats1$^{f/+}$;Lats2$^{f/f}$; YAP/TAZ(mut/WT)* mice.

Sex	TTN	Life span (days)
$H7;L1^{f/+};L2^{f/f};Y^{f/f};T^{f/f}$		
M	2	193
F	2	147
F	2	155
F	2	188
F	3	188
M	2	237
F	1	326
M	N.A.	200
M	1	216
M	1	207
M	3	168
M	5	325
$H7;L1^{f/+};L2^{f/f};Y^{f/f};T^{f/+}$		
F	1	90
M	3	165
F	3	143
F	2	143
F	4	108
F	3	115
M	3	145
F	3	162
F	2	159
M	2	162
F	2	162
$H7;L1^{f/+};L2^{f/f};Y^{f/+};T^{f/f}$		
F	3	142
F	1	126
F	2	148
M	2	188
M	2	126
M	2	188
M	3	256
F	3	200
F	1	93
F	1	156
M	4	295
F	1	170

Sex	TTN	Life span (days)
$H7;L1^{f/+};L2^{f/f};Y^{f/+};T^{f/f}$		
F	4	175
F	2	230
F	2	143
F	1	193
M	2	172
M	4	125
M	1	205
F	2	140
F	2	189
F	3	165
M	5	252
F	4	223
F	3	133
M	2	224
M	2	224
M	5	250
F	2	198
F	2	165
F	1	170
F	2	266
F	9	251
$H7;L1^{f/+};L2^{f/f};Y^{f/+};T^{f/+}$		
F	6	118
F	3	118
M	1	86
F	7	145
F	5	145
F	5	100
M	4	125
M	3	123
M	N.A.	157
F	5	173
F	1	127
F	8	135
F	4	135
M	6	150
M	15	183

Sex	TTN	Life span (days)
$H7;L1^{f/+};L2^{f/f};Y^{f/f};T^{+/+}$		
F	15	116
M	14	134
F	6	98
M	6	109
F	10	166
M	6	127
F	7	136
M	4	147
F	2	73
M	7	161
M	3	153
F	7	108
M	4	189
F	4	131
F	6	137
M	3	124
M	4	137
F	7	127
F	4	145
M	7	145
F	1	115
F	10	106
$H7;L1^{f/+};L2^{f/f};Y^{+/+};T^{f/f}$		
M	4	150
M	4	145
M	1	174
F	1	134
F	4	119
M	1	121
M	2	170
M	1	121
F	1	134
M	3	129
F	2	120
F	1	147
M	1	173
M	1	199

Sex	TTN	Life span (days)
$H7;L1^{f/+};L2^{f/f};Y^{+/+};T^{f/f}$		
F	4	158
F	4	161
M	3	157
F	5	177
F	1	189
M	3	201
M	2	213
M	2	245
F	2	152
F	5	160
$H7;L1^{f/+};L2^{f/f}; Y/T^{WT}$		
F	N.A.	147
F	8	127
M	7	172
M	9	156
F	10	107
F	5	107
F	7	101
F	10	134
F	7	112
F	2	112
M	5	112
F	8	100
F	12	166
M	5	147
F	12	154
F	18	154
M	11	150
F	9	169
F	8	120
M	16	154
M	15	148
M	9	134
F	10	161
F	8	154

F: Female. M: Male. No.: Number. H7: Hoxb7-Cre. L1: Lats1. L2: Lats2. Y: YAP. T: Taz. WT: Wild Type. TTN: Total palpable or visible tumor number. N.A.: Not Available.

Supplemental Table 2. Genotype and phenotype of *H7;Lats1$^{f/f}$;Lats2$^{f/+}$;YAP/TAZ(mut/WT)* mice.

Sex	TTN	Life span (days)	Sex	TTN	Life span (days)	Sex	TTN	Life span (days)	Sex	TTN	Life span (days)
H7;L1f/f;L2f/+;Yf/f;Tf/f			*H7;L1f/f;L2f/+;Yf/f;Tf/+*			*H7;L1f/f;L2f/+;Yf/f;T+/+*			*H7;L1$^{f/f}$;L2$^{f/+}$;Y/T^{WT}*		
F	4	178	M	5	108	F	8	103	F	14	122
F	2	147	M	8	135	F	5	103	F	N.A.	76
M	3	233	M	6	127	M	13	145	F	15	131
F	1	106	M	12	139	M	14	95	F	12	100
M	3	142	F	6	111	F	10	134	M	27	95
M	3	139	F	3	103	F	11	123	F	6	72
M	6	151	*H7;L1f/f;L2f/+;Yf/+;Tf/f*			M	17	141	M	N.A.	122
M	2	126	M	8	236	M	4	85	M	44	122
M	9	196	M	3	135	M	13	93	M	14	130
F	3	159	F	10	58	F	3	74	F	N.A.	122
M	4	187	F	2	159	F	14	126	F	13	111
M	2	185	M	1	100	F	19	126	F	11	111
F	3	154	F	3	107	M	15	143	M	25	139
M	3	154	M	2	218	F	9	126	F	31	81
F	2	118	F	3	146	F	27	92	M	70	131
H7;L1f/f;L2f/+;Yf/f;Tf/+			M	4	147	F	21	92	F	31	89
F	7	109	M	5	104	M	11	97	M	30	123
M	6	128	*H7;L1f/f;L2f/+;Yf/+;Tf/+*			F	11	115	F	24	125
F	4	140	F	12	122	M	15	145	F	27	116
M	3	126	M	11	122	*H7;L1f/f;L2f/+;Y+/+;Tf/f*			F	13	159
M	5	107	F	5	93	F	12	227	M	25	120
M	4	120	F	6	147	F	7	138	M	18	152
M	4	127	M	28	138	M	3	157	F	12	105
M	3	133	M	8	145	F	3	99	F	11	88
M	8	155	M	8	109	M	2	146	F	7	104
F	6	156	*H7;L1f/f;L2f/+;Yf/f;T+/+*			M	6	175			
M	16	147	M	18	80	M	4	146			
F	8	117	M	14	129	M	11	144			
F	5	118	F	19	107	M	7	143			

F: Female. M: Male. No.: Number. H7: Hoxb7-Cre. L1: Lats1. L2: Lats2. Y: YAP. T: Taz. WT: Wild Type. TTN: Total palpable or visible tumor number. N.A.: Not Available.
